# HIF-1α enhances ferroptosis resistance in anaplastic thyroid carcinoma by suppressing ACSL4-mediated lipid metabolic homeostasis

**DOI:** 10.1186/s11658-026-00973-1

**Published:** 2026-06-27

**Authors:** Renjie Xie, Ruixue Geng, Yuchen Wang, Chenyue Zhan, Xinyue Deng, Yanting Duan, Juyong Liang, Jiafeng Wang, Ruimin Liang, Jingyan Ge, Minghua Ge, Xiaozheng Zhu

**Affiliations:** 1 Zhejiang Key Laboratory of Bioorganic Synthesis, College of Biotechnology and Bioengineering, Zhejiang University of Technology, Hangzhou, China; 2 Otolaryngology and Head and Neck Center, Cancer Center, Department of Head and Neck Surgery, Zhejiang Provincial People’s Hospital (Affiliated People’s Hospital), Hangzhou Medical College, Hangzhou, China; 3 Zhejiang Key Laboratory of Precision Medicine Research On Head and Neck Cancer, Zhejiang Provincial People’s Hospital, Hangzhou, China; 4 Zhejiang Provincial Clinical Research Center for Head and Neck Cancer, Zhejiang Provincial People’s Hospital, Hangzhou, China; 5 Department of Thyroid and Breast Surgery, Zhejiang Provincial People’s Hospital Bijie Hospital, Bijie, China; 6 State Key Laboratory of Green Chemical Synthesis and Conversion, Zhejiang University of Technology, Hangzhou, China

**Keywords:** ATC, HIF-1α, ACSL4, Ferroptosis, Lipid metabolism

## Abstract

**Background:**

Anaplastic thyroid carcinoma (ATC) exhibits extreme malignancy with a median survival of less than 6 months. Traditional therapeutic approaches yield limited efficacy, necessitating the urgent identification of novel treatment strategies. The tumor hypoxic microenvironment serves as a key driver of ATC progression and drug resistance, in which the transcription factor hypoxia-inducible factor 1α (HIF-1α) orchestrates key processes in regulating tumor metabolism, immune evasion, and resistance to cell death. Ferroptosis is a novel iron-dependent form of programmed death, defined by excessive peroxidation of polyunsaturated fatty acid phospholipids (PUFA-PL) within cellular membranes.

**Methods:**

In this study, cellular and xenograft models were employed to demonstrate that hypoxia confers ferroptosis resistance to ATC cells. Lipid metabolomics analysis revealed HIF-1α regulates lipid metabolism, and acyl-CoA synthase 4 (ACSL4) was identified as key lipid metabolism-related candidate. Chromatin immunoprecipitation (ChIP) and dual-luciferase reporter assays were used to assess the binding of HIF-1α to the hypoxia-response element (HRE) within the ACSL4 promoter region. Flow cytometric analysis was performed to investigate how HIF-1α inhibition augments the antitumor immunogenicity of PD-1 blockade, as evidenced by enhanced intratumoral CD8^+^ T-cell infiltration and cytokine secretion.

**Results:**

This study identifies a key mechanism by which HIF-1α provides ferroptosis resistance in ATC under the intrinsically hypoxic tumor microenvironment. HIF-1α directly binds the HRE within the ACSL4 promoter, transcriptionally repressing ACSL4 and consequently curtailing PUFA-PL biosynthesis, thereby conferring ferroptosis resistance on ATC cells. In addition, combined treatment with HIF-1α inhibitor and PD-1 blockade effectively suppresses tumor progression and enhances intratumoral CD8^+^ T-cell infiltration.

**Conclusions:**

This study elucidates the molecular mechanism by which HIF-1α mediates anti-ferroptosis in ATC through regulating lipid metabolism and proposes a promising therapeutic strategy in which HIF-1α inhibition acts synergistically with PD-1 blockade for the treatment of ATC.

**Graphical abstract:**

**Figure:**
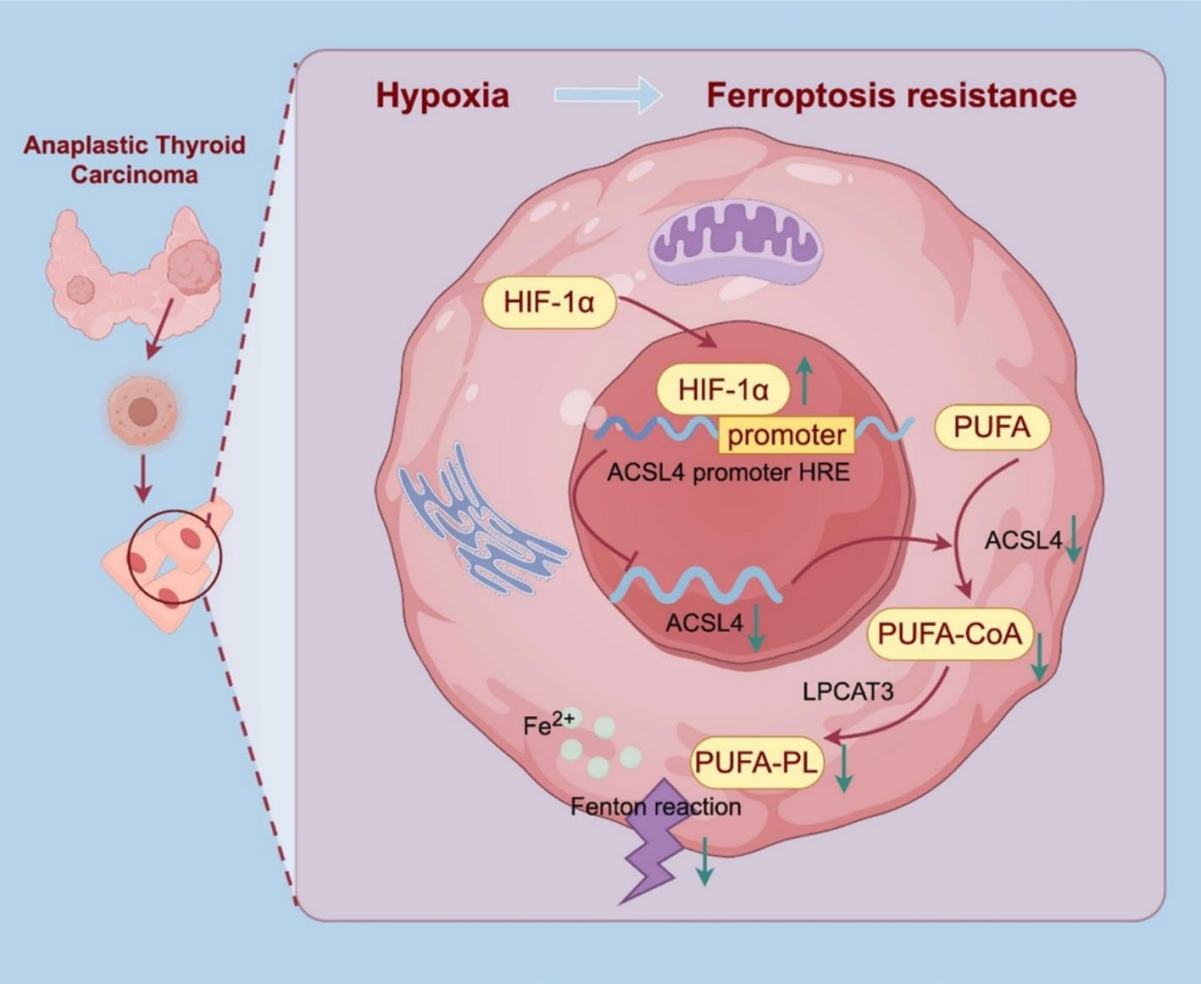

**Supplementary Information:**

The online version contains supplementary material available at 10.1186/s11658-026-00973-1.

## Introduction

Thyroid carcinoma is a malignant tumor derived from thyroid follicular epithelium, with its incidence steadily rising in recent years. Anaplastic thyroid carcinoma (ATC) is the most malignant subtype of thyroid carcinoma, with a median survival of less than 6 months, accounting for over 60% of thyroid-cancer-related mortality [1]. It typically affected the elderly and is characterized by rapid progression, extensive local invasion, and early distant metastasis [2]. Thanks to traditional treatments yielding unsatisfactory outcomes, there was an urgent need to identify novel therapeutic targets and develop more effective diagnostic and treatment strategies [3, 4].

Compared with normal non-tumor cells, tumor cells exhibit heightened iron ion demand to sustain their growth. This iron dependency renders tumor cells more susceptible to ferroptosis [5]. Ferroptosis is an iron-dependent form of regulated cell death triggered by lipid peroxidation in the cell membrane and characterized by morphological and molecular features distinct from apoptosis, necrosis, and autophagy [6, 7]. The primary mechanism involves excessive intracellular ferrous ions, which fuels the Fenton reaction to generate hydroxyl radicals to promote peroxidation of polyunsaturated phospholipids, culminating in cell membrane rupture and ultimately cell death [8, 9]. Concurrently, the presence of abundant hydroxyl radicals disrupts redox balance, suppressing the expression of glutathione peroxidase 4 (GPX4) [10]. Morphologically, ferroptosis induces mitochondrial shrinkage, reduced cristae number, and increased membrane density [11]. Ferroptosis induction has become a novel therapeutic approach in oncology, as it can eliminate cancer cells resistant to multiple anticancer agents [12]. A growing body of research indicates that ferroptosis resistance is a key common mechanism underlying the development of chemotherapy resistance in various types of cancer [13–16]. Further research into ferroptosis will facilitate the development of novel, non-traditional therapeutic targets for ATC.

Lipid metabolism influences cellular susceptibility to ferroptosis by regulating intracellular phospholipid composition and lipid peroxidation processes [17, 18]. Ferroptosis is an iron-dependent form of regulated cell death characterized by the accumulation of lipid peroxides [6]. Lipid peroxidation directly damages cell membranes, leading to cellular dysfunction and eventual death [19–23]. The high susceptibility of polyunsaturated fatty acids (PUFA) to free-radical oxidation stems from the low bond dissociation energy of their bis-allylic hydrogens, which easily undergo hydrogen abstraction and initiate chain-propagating lipid peroxidation [24]. Since ferroptosis is triggered by the peroxidation of membrane phospholipids containing PUFAs (rather than free PUFAs alone), enzymes involved in incorporating PUFAs into phospholipids play an irreplaceable role in the ferroptosis process [25].

Hypoxia is a distinguishing hallmark of solid tumors and is closely associated with malignant progression, which includes increased proliferation, angiogenesis, metabolic reprogramming, invasion, metastasis, and decreased radiation and chemotherapy responsiveness [26]. The mechanisms by which hypoxia drives cellular transformation include transcriptional regulation, epigenetic regulation, and modulation of protein translation and post-translational modifications. Among these, transcriptional regulation mediated by hypoxia-inducible factors (HIFs) represents the most critical pathway for cellular sensing and adaptation to hypoxic environments [27]. Previous studies have demonstrated that HIF-1α confers resistance to ferroptosis through metabolic reprogramming, including increased lactate production, cysteine transport via SLC1A1/SLC7A11, and regulation of GPX4. However, the role of HIF-1α in regulating ferroptosis sensitivity through polyunsaturated phospholipid metabolism remains inadequately understood. Elucidating these mechanisms is crucial for advancing targeted cancer therapies and controlling hypoxia-related diseases.

In this experiment, we found that ferroptosis inducers demonstrated promising efficacy in vitro. However, owing to HIF-1α, the therapeutic effect of ferroptosis was diminished under hypoxic conditions in vivo. To explore the mechanisms regulating ferroptosis resistance in ATC cells under hypoxic conditions, we identified the role of HIF-1α induced by the hypoxic environment in promoting tumor-cell ferroptosis resistance using ATC cells and a mouse xenograft model. Furthermore, we investigated that hypoxia confers ferroptosis resistance to tumor cells by diminishing the intracellular abundance of PUFA phospholipids (PUFA-PL). Mechanistically, HIF-1α directly binds to the hypoxia-response element (HRE) sequence within the ACSL4 promoter region to inhibit transcription, which serves as the rate-limiting enzyme for PUFA-PL biosynthesis. Building on these results, we found that treatment with HIF-1α inhibitor sensitizes tumor cells to ferroptosis and enhances the antitumor immunogenicity of PD-1 blockade by promoting intratumoral CD8^+^ T-cell infiltration and cytokine secretion. This approach transforms ATC from an “immunologically cold” tumor to a “hot” tumor, offering new insights for ATC treatment decision-making.

## Materials and methods

### Cell culture and hypoxic exposure

The human-derived ATC cell line 8505C was purchased from Applied Biological Materials Inc., catalog no. T9074. The KHM-5M cell line was purchased from Wuhan Procell Biotechnology Co., Ltd., catalog no. CL-0623. Cells were routinely cultured in Dulbecco’s modified Eagle medium (DMEM) supplemented with 10% fetal bovine serum (FBS) and 1% penicillin–streptomycin at 37 °C in a 5% CO_2_ incubator. For hypoxic culture, cells were transferred to a hypoxic incubator with oxygen concentration adjusted to 1%, with all other conditions identical to normoxic culture.

### Animal experiments

BALB/c nude mice and C57 mice aged 4–6 weeks and weighing 18–22 g were selected and purchased from Shanghai SLAC Laboratory Animal. Mice were housed in a specific pathogen-free (SPF) animal facility maintained at 22–26 °C with 40–60% relative humidity under a 12 h light/dark cycle, receiving sterile feed and drinking water. For subcutaneous xenografting, ATC cells in the logarithmic growth phase were trypsinized, washed twice with phosphate-buffered saline (PBS), and adjusted to a concentration of 5 × 10^7^ cells/mL. A total of 100 μL of cell suspension (containing 5 × 10^6^ cells) was subcutaneously inoculated into the right axillary region. Post-injection, tumor length (*L*) and width (*W*) were measured using a vernier caliper every 2–3 days. Tumor volume was calculated using the formula *V* = 1/2 × *L* × *W*^2^, continuing until volumes reached approximately 100–150 mm^3^ for subsequent drug-treatment experiments.

### Chemically induced hypoxia

Roxadustat (FG-4592) was employed to simulate hypoxic conditions. ATC cells in the logarithmic growth phase were seeded into 5-cm culture dishes at a density of 50,000 cells per well. Following cell attachment, the spent medium was aspirated and replaced with fresh complete medium supplemented with 50 μM FG-4592. The dishes were then incubated at 37 °C in a 5% CO_2_ incubator. Cells were harvested after 24 h for subsequent experiments.

### Ferroptosis inducer treatment

For experiments involving ferroptosis-inducer treatment, control groups, single-agent treatment groups, and combination treatment groups (e.g., FG-4592 combined with ferroptosis inducers) should be included. ATC cells were seeded into 96-well plates, 24-well plates, or 6-well plates, selecting the appropriate culture plate on the basis of experimental objectives and assay parameters. Following cell attachment, the corresponding reagents were added according to the aforementioned groups, followed by incubation at 37 °C in a 5% CO_2_ incubator for the specified duration prior to subsequent assays for cell viability, lipid peroxidation, and other relevant parameters.

### Western blot

Protein extraction and western blot were performed with minor modifications [9]: 10% sodium dodecyl sulfate (SDS)–polyacrylamide gel electrophoresis (PAGE), 20 min at high speed, 5% skimmed milk powder blocking for 1 h, primary antibody incubation overnight at 4 °C, secondary antibody incubation for 1 h, and enhanced chemiluminescence (ECL) development. The antibodies used were HIF-1α, ACSL4, LPCAT3, and β-actin. Uncropped western blots are provided in the Supplementary File.

### Flow cytometry

For lipid peroxidation detection, ATC cells from different treatment groups were collected and washed twice with prechilled PBS, followed by 5 μM C11-BODIPY 581/591 incubation for 30 min. For cell mortality detection, cells pretreated with indicated compounds were washed twice with prechilled PBS and incubated with propidium iodide (PI) staining solution for 15 min. Following incubation, the mean fluorescence intensity was determined using fluorescence-activated cell sorting (FACS).

### Confocal fluorescence microscopy analysis

Lipid peroxidation was assessed using fluorescence microscopy. Cells were seeded in confocal culture dishes, treated, and stained with C11-BODIPY 581/591 reagent. Changes in green fluorescence intensity were observed under confocal microscopy to evaluate lipid peroxidation levels.

### MDA and GSH content detection

The cellular malondialdehyde (MDA) and glutathione (GSH) contents were detected according to the protocols for the MDA assay kit and GSH assay kit (Beyotime products). For MDA detection, absorbance was measured at 532 nm after sample reaction with reagents and MDA content was calculated via standard curve. For GSH detection, absorbance was measured at 412 nm after sample reaction with reagents and GSH content was calculated via standard curve.

### Quantitative reverse transcription polymerase chain reaction (RT–PCR)

Total RNA was extracted from ATC cells across different treatment groups using an mRNA extraction kit. RNA was reverse transcribed into complementary DNA (cDNA) according to the reverse transcription kit protocol. Using the cDNA as template, quantitative PCR (qPCR) reactions were performed with SYBR Green qPCR Master Mix. Primers were synthesized by Beijing Qingke Biotechnology: ACSL4 forward sequence: 5′-CCTCCAAGTAGACCAACG-3′; ACSL4 reverse sequence: 5′-TGAGCCAAAGGCAAGTAG-3′; LPCAT3 forward sequence: 5′-GCAAGGCAAAGTGGGATG-3′; LPCAT3 reverse sequence: 5′-CCAGGCGTTGGTGTTGAT-3′; actin forward sequence: 5′-CATGTACGTTGCTATCCAGGC-3′; actin reverse sequence: 5′-CTCCTTAATGTCACGCACGAT-3′. Relative expression levels of the target gene messenger RNA (mRNA) were calculated using the 2^−ΔΔCt^ method.

### Chromatin immunoprecipitation (ChIP) assay

ChIP measurements were performed using the Beyotime ChIP Kit according to the manufacturer’s instructions. Chromatin samples were immunoprecipitated with normal rabbit IgG as a negative control and with an HIF-1α-specific antibody. Nucleotide sequence sgHIF1α: 5′-AGATGCGAACTCACATTATG-3′; sgNC: 5′-GCACTACCAGAGCTAACTCA-3′.

### Dual luciferase reporter assay

A fragment encompassing the wild-type (WT) ACSL4 promoter was amplified from the ATC cell genome and cloned into a luciferase reporter vector to generate the pGL3-ACSL4-WT construct (GuanNan. co, Ltd, Hangzhou, China). Mutant luciferase reporter vectors carrying HRE 2 and HRE 8 mutations (MUT 2 and MUT 8) within the ACSL4 promoter were generated via site-directed mutagenesis. ATC cells were seeded into 24-well plates at a density of 8000 cells per well. After cell attachment, cells were transfected with pGL3-ACSL4-WT, pGL3-ACSL4-MUT 2, or pGL3-ACSL4-MUT 8 vectors. A total of 6 h post transfection, the medium was replaced with fresh culture medium and cells were incubated for an additional 24 h. Cells were then washed twice with PBS, lysed with cell lysis buffer at room temperature for 15 min, and centrifuged at 12,000 rpm for 15 min at 4 °C. The supernatant was collected, and luciferase activity was measured following the manufacturer’s instructions (Beyotime). Relative luciferase activity was calculated as the ratio of target gene luciferase activity to control gene luciferase activity.

### Cell viability assay

ATC cells were seeded into a 96-well plate at a density of 3000 cells per well and cultured for 24 h to allow cell attachment. Reagents were added according to different treatment groups, with 5–6 replicate wells per group. After incubation, 10 μL CCK-8 reagent was added to each well followed by incubation at 37 °C for 1–4 h. Absorbance was measured at 450 nm wavelength using a microplate reader. Cell viability (%) was calculated as: (Experimental group absorbance − Blank group absorbance)/(Control group absorbance − Blank group absorbance) × 100%. A dose–response curve was plotted with drug concentration on the *x*-axis and cell viability on the *y*-axis to determine the half maximal inhibitory concentration (IC₅₀) value.

### Histopathological analysis

Following cervical dislocation, tumors and major organs were harvested post-mortem. Specimens were fixed in 4% paraformaldehyde for 24 h, followed by routine dehydration, clearing, and paraffin embedding. Sections were prepared at 4 μm thickness. For hematoxylin and eosin (HE) staining, sections underwent dewaxing, graded hydration, hematoxylin staining, eosin staining, dehydration, clearing, and mounting. Examination was conducted under a light microscope. For 4-HNE immunohistochemical staining, sections underwent dewaxing and rehydration followed by antigen retrieval. Blocking was performed with 3% hydrogen peroxide, followed by 5% bovine serum albumin (BSA) blocking. Primary antibody incubation was conducted overnight, with secondary antibody incubation at room temperature the following day. DAB color development was performed, followed by counterstaining with hematoxylin, dehydration, clearing, and mounting. Sections were examined and photographed under a microscope. Image analysis software (ImageJ) was employed to quantify the area of positive staining.

### Transmission electron microscopy

Cells were prefixed with 2.5% glutaraldehyde, postfixed with 1% osmium tetroxide, dehydrated using acetone gradient, embedded in Epon812, sectioned into ultrathin sections of 70 nm, double-stained with uranium-lead, and imaged using an HT7800 camera.

### Lipid metabolomics analysis

ATC cells cultured under varying oxygen concentrations were harvested, washed three times with prechilled PBS, rapidly frozen in liquid nitrogen, and stored at −80 °C for subsequent use. Lipid extraction was performed. The organic phase was transferred to a fresh tube, dried under nitrogen, and resuspended in methanol. Analysis was conducted using a liquid chromatography–mass spectrometry (LC–MS) system. Data acquisition was processed using Xcalibur software. Principal component analysis (PCA) and partial least squares discriminant analysis (PLS-DA) were employed for data analysis to identify differentially expressed metabolites.

### In vitro BMDC maturation and antigen presentation assay

In vitro maturation and antigen presentation experiments were conducted using mouse bone marrow-derived dendritic cells (BMDCs). BMDCs were isolated from C57BL/6J mice, differentiated with GM-CSF and IL-4, seeded in 12-well plates, and treated with either pure culture medium, 8505C cells, or 8505C cells overexpressing ACSL4. After 48 h, the cells were stained with Zombie NIR viability dye, blocked with anti-CD16/32 antibody, and labeled with anti-CD45, CD11c, CD80, CD86, and MHC II antibodies, followed by analysis via flow cytometry. Mature BMDCs were defined as CD80⁺CD86⁺ or MHC II⁺ cells within the CD45⁺CD11c⁺ population.

### Statistical analysis

Experimental data were statistically analyzed using GraphPad Prism 9.0 software. Comparisons between two groups employed Student’s *t* test; comparisons among multiple groups utilized one-way analysis of variance (ANOVA). Where variance homogeneity was established, Tukey’s multiple comparison test was subsequently applied; where variance heterogeneity was present, Dunnett’s T3 test was employed. All data are expressed as mean ± standard deviation (*x* ± *s*), with *P* < 0.05 indicating statistically significant differences.

## Results

### Hypoxia confers ferroptosis resistance in ATC cells

**Fig. 1:**
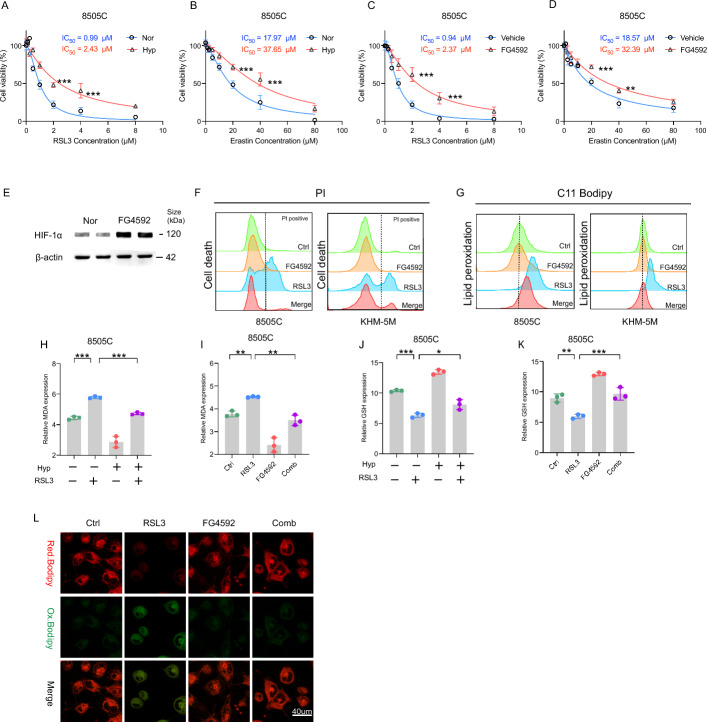
Hypoxia drives ATC cells’ resistance to ferroptosis. **A**–**D** Exposure to 1% oxygen (24 h) or the chemical inducer FG-4592 (50 mM, 24 h) reduced the drug sensitivity of ATC cells to erastin (10 μM, 24 h)/RSL3 (1 μM, 24 h). **E** Western blot analysis validates the upregulation of HIF-1α expression in ATC cells following treatment with the chemical inducer FG-4592 (50 mM, 24 h). Flow cytometry analysis of lipid peroxidation (**F**) and cell death rate (**G**) in ATC cells treated with RSL3 (1 μM, 24 h) under normoxic and hypoxic conditions. Exposure to 1% oxygen (**H**) or chemically induced hypoxia (**I**) attenuates the increase in intracellular malondialdehyde (MDA) levels in ATC cells induced by the ferroptosis inducer RSL3 (1 μM, 24 h) (8505C). A 1% oxygen environment (**J**) or chemically induced hypoxia (**K**) reversed the downregulation of intracellular GSH levels in ATC cells induced by the ferroptosis inducer RSL3 (1 μM, 24 h) (8505C). **L** Lipid peroxidation in ATC cells treated with RSL3 (1 μM, 24 h) under normoxic and hypoxic conditions was visualized via confocal microscopy

Considering that tumor cells exhibit iron addiction and may therefore be intrinsically susceptible to ferroptosis, we exposed ATC cell lines to various ferroptosis inducers for 24 h. Because hypoxia is a defining characteristic of the solid-tumor microenvironment, we compared the sensitivity of these cells with the inducers under standard normoxic (21% O_2_) versus hypoxic (1% O_2_) conditions to determine whether hypoxia alters ferroptosis tolerance [28]. Results demonstrated that hypoxia effectively suppressed ferroptosis inducer (FIN)-induced cell death. Consistent with this, FG4592, a HIF prolyl-hydroxylase inhibitor [29, 30], exhibited similar results, with upregulation of HIF-1α (Fig. 1A–E; Supplementary Fig. 1A–D). This phenomenon likely arises because hypoxia activates a series of adaptive mechanisms within ATC cells, thereby enhancing their resistance to ferroptosis [31, 32]. For lipid peroxidation detection, the lipid peroxidation sensor C11-BODIPY 581/591 reagent was selected [33–35]. For cell mortality detection, propidium iodide (PI) staining was used [36]. We observed that FG4592 significantly inhibits FIN-induced cell death (Fig. 1F). Concurrently, hypoxia attenuated the RSL3-induced increase in lipid peroxidation (Fig. 1G). Subsequently, we examined the ferroptosis markers MDA and GSH [37–39]. The results clearly indicate that hypoxia significantly affects MDA and GSH levels in ATC cells. Under hypoxic conditions, the RSL3-induced increase in MDA levels in ATC cells was abolished (Fig. 1H, I; Supplementary Fig. 1E, F). At the same time, GSH levels in ATC cells were generally elevated under hypoxic conditions (Fig. 1J, K; Supplementary Fig. 1G, H). Furthermore, we examined lipid peroxidation in RSL3-treated ATC cells under normoxic and hypoxic conditions using confocal microscopy. The results were consistent with those detected by flow cytometry (Fig. 1L). At the same time, we found that hypoxia similarly promotes the development of ATC resistance to apoptosis (Supplementary Fig. 1I–L). Taken together, these findings demonstrate that hypoxia promotes resistance to ferroptosis in ATC cells.

### Hypoxia-inducible factor HIF-1α facilitates the development of ATC tumors

**Fig. 2:**
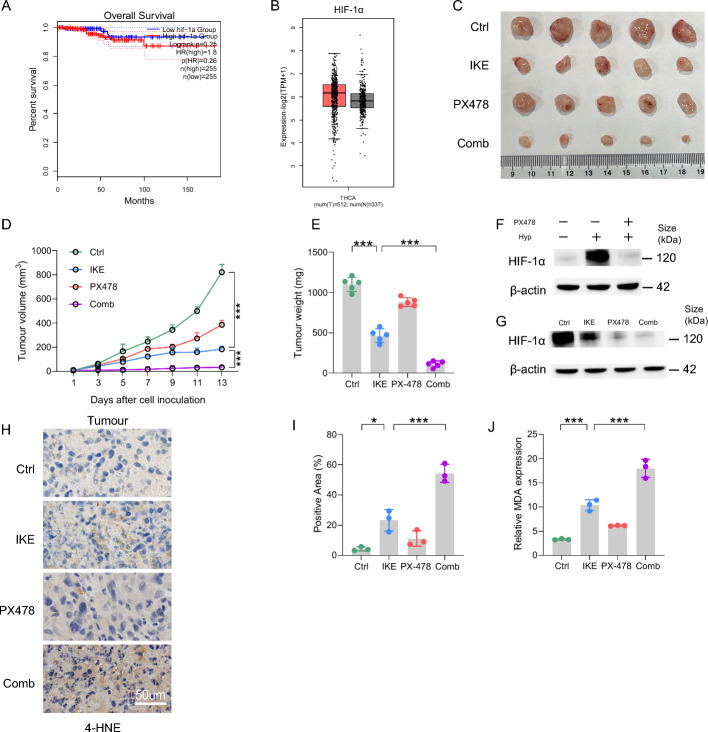
HIF-1α facilitates the development of ATC tumors. **A**, **B** HIF-1α expression correlates with poor prognosis in patients with ATC. Data sourced from The Cancer Genome Atlas (TCGA) database. **C**–**E** Upon tumor volume reaching 100 mm^3^, mice were randomly assigned to four groups (*n* = 5/group): control (saline), IKE group (ferroptosis inducer, 25 mg/kg, intraperitoneal injection, every other day), PX478 group (HIF-1α-specific inhibitor, 25 mg/kg, oral gavage, every other day), and PX478 + IKE combination group (same dosages as single-agent groups). PX478 potentiated IKE’s suppression of ATC xenografts, reducing tumor volume (**C**, **D**) and weight (**E**). **F** Validation that PX-478 (15 μM, 24 h) induces intracellular HIF-1α degradation in ATC cells. **G** Validation of tumor hypoxia via western blot analysis. **H** 4-HNE immunohistochemical staining of mouse tumors and HE staining of visceral tissue sections from tumor-bearing mice. **I** PX478 potentiates IKE treatment in reducing tumor MDA content in ATC xenografts. **J** PX478 increases 4-HNE-positive area in ATC xenografts following IKE treatment

Analysis of prognostic relevance in clinical samples is a core method for validating the clinical value of molecular biomarkers. To clarify the expression characteristics and prognostic significance of HIF-1α in thyroid cancer, clinical data from online databases revealed that HIF-1α was highly expressed in tumors and positively associated with poor prognosis in patients with thyroid cancer (Fig. 2A, B). Building on the association between HIF-1α and poor ATC prognosis in clinical samples, the study further validated the functional role of HIF-1α in ATC progression using a nude mouse xenograft model. This study used the oral HIF-1α inhibitor PX478 [40–42] in combination with imidazole ketone erastin (IKE). We found that monotherapy effectively slowed tumor growth but the combination therapy group maximally reduced tumor growth rate and tumor weight (Fig. 2C–E). No significant differences in body weight were observed among groups during the experiment, and multi-marker serum assays revealed no differences between experimental and blank control groups (Supplementary Fig. 1A–C). Concurrently, HE-stained sections of major visceral organs (heart, liver, spleen and kidney) in tumor-bearing mice revealed no significant pathological damage in any group (Supplementary Fig. 2), indicating the combined treatment regimen possesses favorable safety. Validation of PX478’s efficacy on ATC cells demonstrated its ability to effectively suppress hypoxia-induced upregulation of HIF-1α expression (Fig. 2F). We then examined the expression of HIF-1α within the tumor tissue. Results further confirmed that PX478 effectively suppressed HIF-1α expression activity within ATC xenografts (Fig. 2G). Lipid peroxidation levels and histopathological analysis were performed on tumor tissues at the experimental endpoint. We observed minimal 4-HNE-positive staining areas in tumor tissues from the control and PX478 monotherapy groups, whereas the combination therapy group exhibited significantly enlarged 4-HNE-positive regions with markedly enhanced staining intensity (Fig. 2H, I). Detection of MDA [43] levels in tumor tissues revealed significantly higher MDA content in the tumor tissues of the combination group compared with the IKE monotherapy group (Fig. 2J). These results suggest that HIF-1α expression is closely associated with ATC tumor progression and may participate in ATC development by regulating malignant biological behaviors of tumor cells.

### Hypoxia inhibits tumor-cell ferroptosis by suppressing lipid peroxidation accumulation

**Fig. 3:**
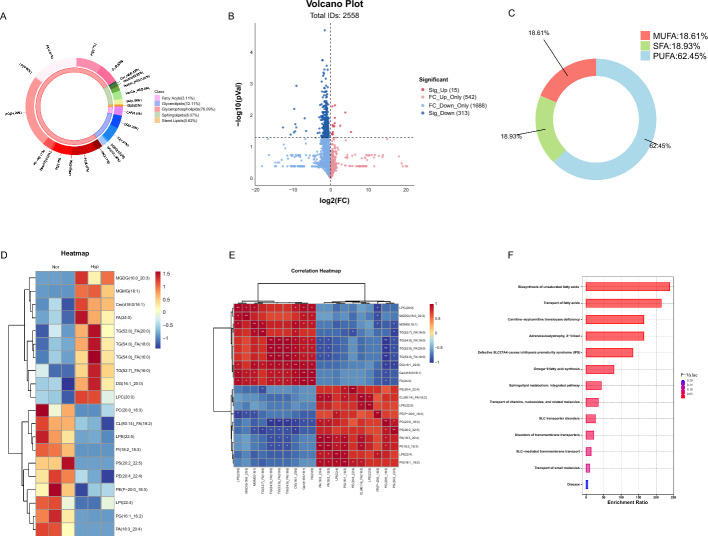
Hypoxia inhibits tumor cell ferroptosis by suppressing lipid peroxidation accumulation. **A** Ring diagram comparing lipid composition differences under varying oxygen conditions. **B** The volcano plot displays the metabolic state of lipids. **C** Composition of glycerophospholipids. **D** Representative upregulated and downregulated metabolites. **E** Heat map correlating differentially expressed metabolites in ATC cells. **F** Bar chart displaying enriched metabolic pathways for differential metabolites between normoxic and hypoxic ATC cells

Lipid peroxidation constitutes a pivotal event in ferroptosis, with phospholipid peroxidation representing the core and critical form of lipid peroxidation within this process [20–25]. To comprehensively characterize lipid profiles in our samples, we conducted lipid metabolomics analysis of ATC cells under varying oxygen concentrations. Circular plot (Supplementary Fig. 3A) reveals samples were rich in glycerophospholipids, sphingolipids, sterol lipids, and glycerides. Among these, glycerophospholipids exhibited the most pronounced oxygen-concentration-dependent variation, accounting for 76.09% (Fig. 3A). Furthermore, we visualized the statistical analysis of differentially expressed metabolites using a volcano plot. The results indicate that most lipids are downregulated under hypoxic conditions (Fig. 3B). Simultaneously, we observed a decrease in lipid unsaturation under hypoxic conditions (Supplementary Fig. 3B). Analysis of glycerophospholipid composition revealed that PUFA-PL constituted 62.45% of the profile (Fig. 3C). On the basis of the above analysis, we screened and presented representative significantly up- and down-regulated substances among differentially expressed compounds (Fig. 3D). Box plots visually demonstrated the significant downregulation trend of PUFA-PL (Supplementary Fig. 3C). Subsequently, we performed differential metabolite correlation analysis across various lipid metabolites. Results revealed positive correlations among PUFA-PL metabolite expressions (Fig. 3E). This indicates that PUFA-PL downregulation in the hypoxia group is not coincidental but reflects suppressed intracellular PUFA-PL synthesis under hypoxic conditions. Complex metabolic reactions and their regulation within organisms do not occur in isolation; they are often coordinated by intricate pathways and networks composed of multiple genes and proteins. The observed metabolic differences may be linked to processes such as unsaturated fatty acid biosynthesis and fatty acid transport (Fig. 3F). This finding points the way for subsequent research, where we will delve deeper into how these pathways specifically contribute to hypoxia-induced changes in PUFA-PL metabolism.

### HIF-1α suppresses ferroptosis by regulating ACSL4

**Fig. 4:**
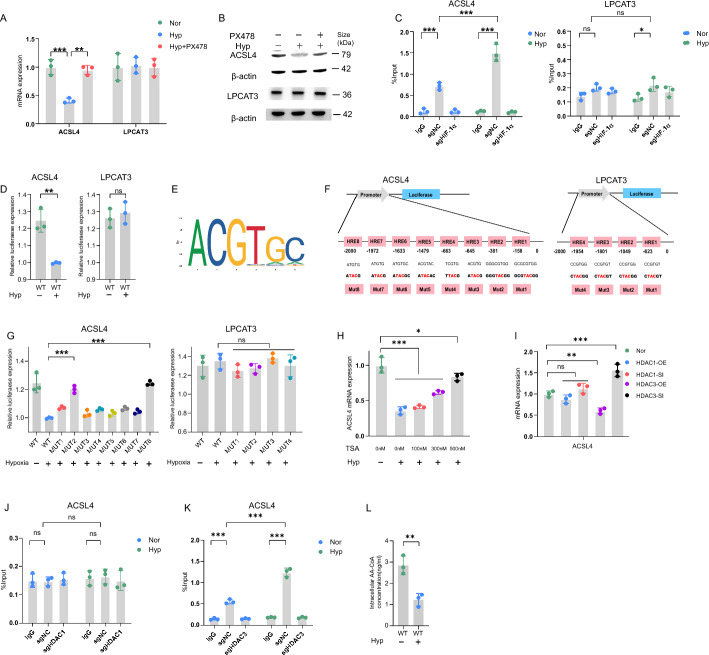
HIF-1α suppresses ferroptosis by regulating *ACSL4*. **A** Bar chart of *ACSL4* and *LPCAT3* mRNA expression levels in 8505C cells under normoxia, hypoxia, and hypoxia + PX478 conditions. **B** Western blot analysis of ACSL4 and LPCAT3 protein expression in 8505C cells under normoxia, hypoxia, and hypoxia + PX478 conditions. **C** ChIP assay determining HIF-1α transcriptional activity within the *ACSL4* and *LPCAT3* promoter region. **D** HIF-1α expression modulates luciferase activity driven by the *ACSL4* and *LPCAT3* promoter. **E** Schematic of the conserved HRE sequence (5′-RCGTG-3′) recognized by HIF-1α. **F** Schematic of *ACSL4* and *LPCAT3* promoter HRE site mutation vector construction. **G** A dual-luciferase reporter assay to assess the promoter activity of different HRE-mutated vectors (*ACSL4* and *LPCAT3*) under hypoxic conditions. **H** qRT–PCR analysis of *ACSL4* mRNA expression in cells treated with the histone deacetylase (HDAC) inhibitor TSA under normoxic or hypoxic conditions. **I** qRT–PCR analysis of *ACSL4* mRNA expression in cells with overexpression (OE) or knockdown (SI) of HDAC1 or HDAC3, compared with the normal (Nor) control. **J**, **K** ChIP–qPCR analysis of HDAC1 and HDAC3 binding to the *ACSL4* promoter under normoxia (Nor) or hypoxia (Hyp). **L** Intracellular enzyme-linked immunosorbent assay (ELISA) analysis of AA-CoA, the catalytic product of *ACSL4*’s preferred substrate AA

Within the ferroptosis-associated lipid metabolism pathways, ACSL4 and LPCAT3 serve as key rate-limiting enzymes for the generation of PUFA-PL [44–47]. Previous studies have demonstrated that elevated HIF-1α expression promotes ferroptosis resistance in tumor cells; however, whether it directly regulates the expression of lipid metabolism-related genes *ACSL4* or *LPCAT3* remains unclear. qPCR was employed to detect mRNA expression levels of both enzymes under varying oxygen concentrations, analyzing their correlation with HIF-1α. The results demonstrated that hypoxia significantly downregulated *ACSL4* mRNA, a process reversible by PX478. Conversely, *LPCAT3* expression remained unchanged (Fig. 4A). Western blot validation confirmed this consistency (Fig. 4B), suggesting *ACSL4* may represent a potential downstream target of HIF-1α. To further determine whether HIF-1α binds the *ACSL4* and *LPCAT3* promoter region, ChIP experiments were applied and results showed that in the hypoxia-treated group, the enrichment level of the *ACSL4* promoter region in chromatin precipitated by anti-HIF-1α antibody was significantly higher than in the IgG control group. Simultaneously, HIF-1α knockout abolished *ACSL4* promoter region enrichment, indicating binding between HIF-1α and *ACSL4* promoter (Fig. 4C), whereas no obvious change was detected in the *LPCAT3* group. Dual luciferase activity results demonstrated that in transfected cells, the luciferase activity of pGL3-ACSL4-pro under normoxic conditions was significantly higher than that in cells under hypoxic conditions (Fig. 4D). Meanwhile, parallel *LPCAT3* assays showed that hypoxia did not significantly alter the binding of HIF-1α to its promoter region nor did it affect luciferase activity, suggesting that *LPCAT3* is not a direct transcriptional target of HIF-1α. As HIF-1α primarily recognizes HRE (5′-RCGTG-3′) within the promoters of its target genes (Fig. 4E) [48], we analyzed the HRE-binding sites within the promoter regions of *ACSL4* and *LPCAT3* and identified candidate HRE sites (Fig. 4F). Hypoxia markedly suppressed luciferase activity from the wild-type (WT) *ACSL4* promoter, whereas mutations in HRE 2 and HRE 8 (MUT 2 and MUT 8) significantly enhanced luciferase activity under hypoxia, indicating HRE 2 and HRE 8 as potential HIF-1α binding sites within the *ACSL4* promoter region (Fig. 4G). Analysis of the *LPCAT3* promoter revealed no functional HIF-1α response elements in its promoter region, further confirming that *LPCAT3* is not directly regulated by HIF-1α.

HIF-1α has traditionally been widely regarded as a transcriptional activator rather than a transcriptional repressor. This study found that HIF-1α can mediate the transcriptional repression of *ACSL4*, suggesting that synergistic transcriptional repressors may be involved in regulating this process. Previous studies have shown that HIF-1α can recruit histone deacetylases (HDACs) to form transcriptional repression complexes [49]. As a broad-spectrum HDAC inhibitor, we found that TSA reverses hypoxia-induced *ACSL4* mRNA suppression in a dose-dependent manner, confirming the involvement of HDACs in this process (Fig. 4H). In the nucleus, HIF-1α primarily interacts with HDAC1 and HDAC3, both of which are its classic co-regulators [49]. In this study, we prioritized screening these two factors, and the results showed that HDAC3, not HDAC1, participates in HIF-1α-mediated *ACSL4* transcriptional repression (Fig. 4I). ChIP analysis demonstrated that hypoxia-induced HIF-1α specifically binds to the *ACSL4* promoter in an HDAC3-dependent manner, while HDAC1 did not show significant binding to the *ACSL4* promoter (Fig. 4J, K). ACSL4 catalyzes the conversion of arachidonic acid (AA)/eicosapentaenoic acid (EPA) and other long-chain PUFAs into acyl-CoA, representing a key activation step in unsaturated fatty acid synthesis [47]. Following construction of siACSL4 and ACSL4 overexpression (ACSL4-OE) ATC cell lines (Supplementary Fig. 4A, B), addition of α-linolenic acid (ALA), EPA, docosahexaenoic acid (DHA), docosapentaenoic acid (DPA), linoleic acid (LA), γ-linolenic acid (GLA), dihomo-γ-linolenic acid (DGLA), or AA to culture medium reduced cell viability for all eight PUFAs, with AA exhibiting the strongest effect. In addition, ACSL4-OE exacerbated toxicity, indicating AA as its preferred substrate (Supplementary Fig. 4C, D). Gradient AA-CCK-8 assays revealed significantly reduced viability in ACSL4-OE versus negative control (NC), while siACSL4 groups showed significantly higher viability than NC (Supplementary Fig. 4E–H), confirming ACSL4 expression levels determine ATC sensitivity to AA. To elucidate the death mechanism, interventions with Lip-1, Nec-1, and ZVAD were employed. Only the ferroptosis inhibitor Lip-1 substantially reversed ACSL4-OE-induced death (Supplementary Fig. 4I, J), demonstrating that exogenous AA intake renders cells sensitive to ferroptosis. To clarify the causal relationship between the HIF-1α–ACSL4 axis and alterations in lipid metabolism, we measured the intracellular levels of AA-CoA, the product of AA catalyzed by ACSL4. The results showed that hypoxia significantly reduced intracellular AA-CoA levels, confirming that HIF-1α reduces PUFA-PL synthesis by inhibiting the enzymatic function of ACSL4 (Fig. 4L). Collectively, these results suggest HIF-1α dynamically regulates intracellular lipid peroxidation substrates by modulating ACSL4 expression, thereby balancing ferroptosis susceptibility and survival requirements in tumor cells.

### Overexpression of ACSL4 abrogates the ferroptosis resistance conferred by HIF-1α

**Fig. 5:**
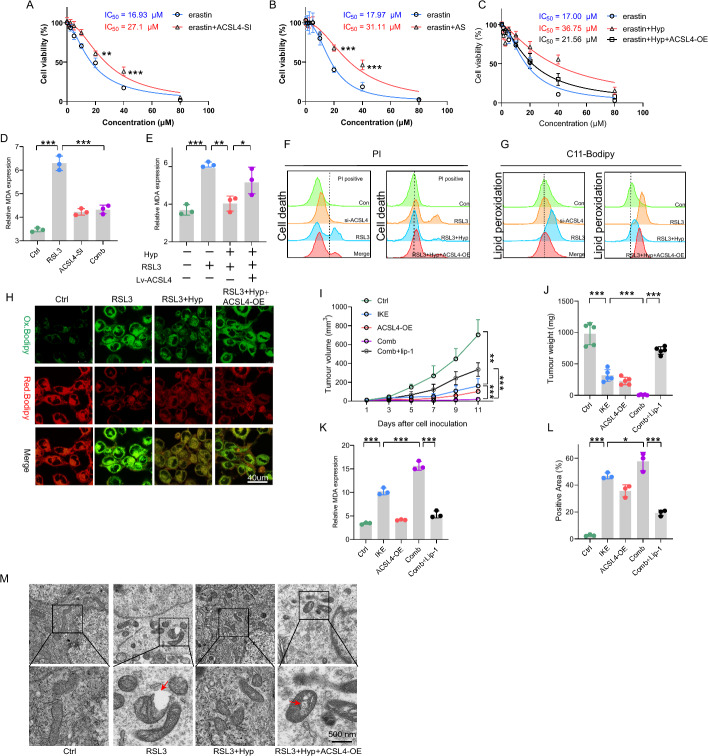
Overexpression of ACSL4 abrogates the ferroptosis resistance conferred by HIF-1α. **A**–**C** Gradient erastin killing curves and IC_50_ statistics: ACSL4-OE significantly shifts the curve downward, reversing the hypoxic survival advantage; siACSL4 or pharmacological inhibition (AS-252424) shifts the curve upward, indicating enhanced resistance. **D**–**E** Quantification of lipid peroxidation end-product MDA: following RSL3 stimulation, hypoxia reduced MDA levels, with ACSL4-OE completely counteracting hypoxia-induced suppression; siACSL4 attenuated MDA elevation. **F** PI probe flow cytometry revealed hypoxia + siACSL4 dual conditions markedly reduced mortality, whilst ACSL4-OE eliminated hypoxia effects and increased cell death rates. **G** C11-BODIPY 581/591 flow cytometry revealed ACSL4-OE shifted the lipid reactive oxygen species (ROS)-positive peak to the right, abolishing hypoxic protection; siACSL4 shifted it to the left, indicating reduced peroxidation. **H** Confocal imaging revealed green fluorescence representing lipid peroxidation-positive signals. Hypoxia and siACSL4 attenuated green fluorescence, whereas ACSL4-OE restored fluorescence intensity by counteracting hypoxia effects. **I** Upon tumor volume reaching 100 mm^3^, mice were divided into five groups (*n* = 6/group): NC group (saline), ACSL4-OE group, IKE group (ferroptosis inducer, 25 mg/kg, intraperitoneal injection, every 2 days), ACSL4-OE + IKE group (same dosage as IKE group), and ACSL4-OE + IKE + Lip-1 group (Lip-1 being a ferroptosis inhibitor, 10 mg/kg, co-administered with IKE). In the mouse tumor model, ACSL4 overexpression enhanced IKE’s therapeutic effect on tumors, whilst Lip-1 attenuated the combined treatment efficacy. **J** Bar chart of terminal tumor weights in nude mouse ATC xenograft experiments, consistent with growth curve results. **K**–**L** Intratumor oxidative damage outcomes: MDA content and 4-HNE immunohistochemical positive areas peaked in the ACSL4-OE + IKE group, with Lip-1 reversal. **M** Mitochondrial electron microscopy images reveal RSL3-induced cristae disruption and increased membrane density; hypoxia alleviates damage; ACSL4-OE eliminates hypoxia effects, reinstating cristae loss and matrix condensation

ACSL4 is a central lipid metabolic switch governing ferroptosis susceptibility [44]. To further demonstrate that HIF-1α in the hypoxic microenvironment of ATC tumors suppresses ACSL4 transcription, thereby reducing PUFA-PL synthesis and conferring ferroptosis resistance to tumor cells, we employed ACSL4 overexpression to investigate whether it could abolish hypoxia-induced ferroptosis resistance. The results showed that ACSL4-OE increased the sensitivity of ATC cells to FIN. Conversely, siACSL4 or pharmacological inhibition (AS-252424) markedly enhanced cellular resistance to ferroptosis (Fig. 5A–C). To further validate this finding, intracellular MDA levels were measured, complemented by flow cytometry and confocal microscopy to assess lipid peroxidation and cell death rates. We found that ACSL4-small interfering RNA (siRNA) markedly attenuated RSL3-induced increases in intracellular MDA levels (Fig. 5D). Hypoxia downregulated RSL3-induced MDA elevation, whereas ACSL4-OE abolished this effect (Fig. 5E). Subsequent flow cytometric analysis revealed that revealing that ACSL4-OE reversed the protective effect of hypoxia on ATC cells, whereas ACSL4-siRNA mitigated RSL3-induced cell death (Fig. 5F). Consistently, both hypoxia and ACSL4-siRNA reduced the proportion of lipid peroxidation-positive cells, while ACSL4-OE eliminated the hypoxia-induced decrease in this proportion (Fig. 5G). To visually assess lipid peroxidation distribution, confocal microscopy fluorescence imaging was performed on RSL3-treated cells. Results demonstrated that hypoxia and ACSL4-siRNA markedly attenuated RSL3-induced lipid peroxidation, whereas ACSL4-OE reversed the hypoxic attenuation of RSL3-induced lipid peroxidation (Fig. 5H; Supplementary Fig. 5A). In mouse xenograft experiments, the ACSL4-OE + IKE group exhibited the smallest tumor volume and weight within its cohort. Supplementation with Lip-1 restored tumor growth rate and final weight (Fig. 5I, J; Supplementary Fig. 5B). Safety assessments revealed no differences in body weight among groups (Supplementary Fig. 5C). Endpoint analyses demonstrated the highest tumor MDA levels in the ACSL4-OE + IKE group, alongside significantly increased 4-HNE immunopositive areas. Lip-1 supplementation reversed these alterations, confirming ACSL4’s role in promoting lipid peroxidation and enhancing ferroptosis activation in vivo (Fig. 5K, L). As previously noted, a distinctive hallmark of ferroptosis is altered mitochondrial morphology, including reduced volume, decreased cristae number, and increased membrane density. The results showed hypoxia ameliorated RSL3 induced cristae disruption and increased membrane density. ACSL4-OE eliminated hypoxia’s protective effect on cellular mitochondria, providing subcellular-level evidence for these findings (Fig. 5M). Consistent with this, ACSL4 overexpression alone could induce mitochondrial ferroptosis characteristics (Supplementary Fig. 5D). Taken together, ACSL4 overexpression overrides HIF-1α-mediated protection in tumor cells, abolishing HIF-1α-conferred ferroptosis resistance.

### PX478 enhances PD-1 blockade efficacy by promoting tumor-infiltrating T cell immune responses

**Fig. 6:**
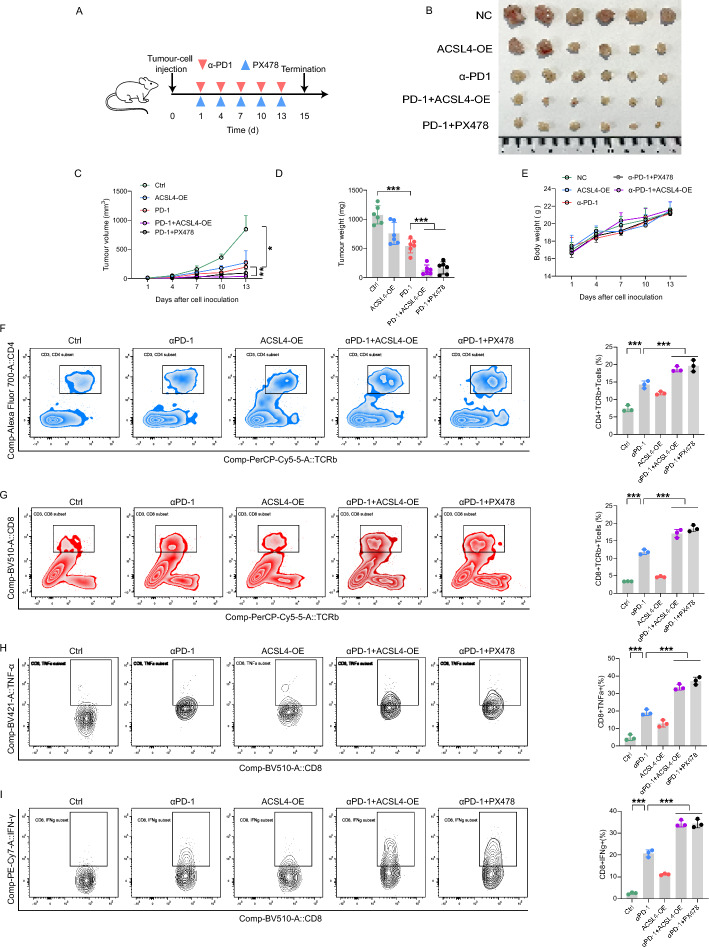
PX478 enhances PD-1 blockade efficacy by promoting tumor-infiltrating T cell immune responses. **A** Timeline diagram of the therapeutic regimen for C57 mice with transplanted ATC tumors. **B** Upon tumor volume reaching 100 mm^3^, mice were divided into five groups (*n* = 6 per group): negative control (NC), ACSL4 overexpression (ACSL4-OE), PD-1 antibody (PD-1), combination of PD-1 antibody and ACSL4 overexpression (PD-1 + ACSL4-OE), and combination of PD-1 antibody and PX478, an HIF-1α inhibitor (PD-1 + PX478). Tumors were excised and photographed at the end of the experiment. **C** Growth curve of C57 mouse ATC xenografts. **D** Bar chart of terminal tumor weights in C57 mice with ATC xenografts. **E** Line graph of body-weight changes in each group during the C57 mouse ATC xenograft experiment. **F** Flow cytometry-quantified bar chart of CD4^+^TCRβ^+^ T-cell infiltration proportion within C57 mouse ATC xenografts. **G** Flow cytometry-quantified bar chart of CD8^+^TCRβ^+^ T-cell infiltration proportion within C57 mice ATC xenografts. **H** Flow cytometry-quantified bar chart of TNF-α^+^CD8^+^ T cell proportion within C57 mice ATC xenografts. **I** Flow cytometry quantitative bar chart of IFN-γ^+^CD8^+^ T-cell proportion within C57 mice ATC-transplanted tumors

Immune checkpoint inhibitor therapy, particularly anti-PD-1 therapy, has revolutionized cancer treatment. However, objective response rates remain limited, and PD-1 monotherapy has shown limited efficacy against ATC tumors; therefore, there is an urgent need to develop strategies to improve treatment outcomes [50–54]. Here, we investigated the interaction between ACSL4, HIF-1α, and PD-1 blockade in a mouse tumor model. Mouse tumor cells were subcutaneously inoculated into mice, and treatments were administered every other day (Fig. 6A). Tumor volumes were significantly smaller in the PD-1 + ACSL4-OE and PD-1 + PX478 groups compared with the NC, ACSL4-OE, and PD-1 monotherapy groups, clearly demonstrating tumor size reduction (Fig. 6B). Dynamic monitoring of tumor volume from day 7 revealed significantly slower growth rates in the PD-1 + ACSL4-OE and PD-1 + PX478 groups compared with other groups (Fig. 6C). Data showed that tumors in the PD-1 + ACSL4-OE and PD-1 + PX478 groups were significantly lighter than those in other groups, with a statistically significant difference compared with the PD-1 antibody monotherapy group (Fig. 6D). Concurrently, mouse body-weight charts (Fig. 6E) demonstrated that body weights remained relatively stable throughout the experiment across all treatment groups. In addition, HE staining of major organs (heart, liver, spleen, and kidney) collected post experiment revealed normal cellular morphology and intact tissue structure in all mice. No significant pathological alterations—including cellular degeneration, necrosis, or inflammatory cell infiltration—were observed (Supplementary Fig. 6A). Regarding tumor-infiltrating CD4^+^ T and CD8^+^ T cells, flow cytometry analysis revealed significantly higher proportions of CD4^+^ TCRβ^+^ T and CD8^+^ TCRβ^+^ T cells in the PD-1 + ACSL4-OE and PD-1 + PX478 groups compared with the PD-1 antibody monotherapy group, with statistically significant differences (Fig. 6F, G). These findings collectively demonstrate that ACSL4 overexpression and HIF-1α inhibition effectively promote CD4^+^ T and CD8^+^ T cell infiltration into tumor tissues, providing a cellular correlate for enhancing antitumor immune responses. As cytokines serve as the primary executors of T cell-mediated tumor killing, the results showed that the percentages of TNF-α^+^ and IFN-γ^+^ CD4^+^ T and CD8^+^ T cells in the PD-1 + ACSL4-OE group and PD-1 + PX478 group were significantly higher than in the PD-1 antibody monotherapy group (Fig. 6H, I; Supplementary Fig. 6B, C). Concurrently, analysis of Gzmb^+^ CD8^+^ T cells yielded results consistent with the previously reported TNF-α^+^ and IFN-γ^+^ findings (Supplementary Fig. 6D). To investigate whether ACSL4-mediated ferroptosis directly activates antitumor immunity, we examined the antigen-presenting phenotype of ATC cells. Flow cytometry analysis revealed that, compared with the control group, ACSL4-OE significantly upregulated the expression of the co-stimulatory molecules CD80/CD86 (Supplementary Fig. 6E) and MHC-II (Supplementary Fig. 6F), which indicated that ACSL4-driven lipid metabolic remodeling enhances the immunogenicity of tumor cells by boosting antigen presentation, potentially providing a mechanistic basis for the activation and infiltration of CD8⁺ T cells and laying the groundwork for subsequent cell-mediated immune-induced cell death. Taken together, the above findings suggest that ACSL4 overexpression may promote dendritic cell maturation and antigen presentation, enhancing CD8^+^ T cell immune responses and cytokine release, thereby improving the efficacy of PD-1 therapy.

## Discussion

Ferroptosis is an iron-dependent, lipid peroxidation-driven form of programmed cell death that was discovered in 2012 [6, 7]. One of its key regulatory pathways is the “ACSL4–PUFA-PL–ROS” cascade [22]. In phenotypic experiments, we found that ATC cells exhibited greater resistance to FIN-induced ferroptosis under hypoxic conditions. Using lipid metabolomics, we systematically analyzed the “hypoxia–glycerophospholipid–PUFA” profile of ATC cells. Hypoxia led to a decrease in total glycerophospholipid content, a significant reduction in PUFA-containing lipids, and lower lipid unsaturation (Fig. 3), which directly reduced the cells sensitivity to erastin/RSL3/IKE (Figs. 1, 2). Mechanistically, the HIF-1α–ACSL4 pathway revealed in this study differs significantly from classical HIF-dependent pathways (SLC7A11-GPX4, SLC1A1). Previous studies reported that HIF-1α enhances antioxidant defense by activating SLC1A1 and SLC7A11 or inducing lactate metabolism, whereas this study found that HIF-1α cooperates with HDAC3 to bind to the ACSL4 promoter region, transcriptionally represses ACSL4 to reduce PUFA-PL synthesis, acting at a more upstream stage in the generation of lipid peroxidation substrates. This strategy of undermining the attack confers greater resistance to ferroptosis on tumor cells.

In ferroptosis-related lipid metabolism pathways, ACSL4 and LPCAT3 are key rate-limiting enzymes for PUFA-PL generation. ACSL4 catalyzes the conversion of polyunsaturated fatty acids into acyl-CoA, while LPCAT3 integrates them into phospholipid molecules, jointly providing substrates for lipid peroxidation during ferroptosis [44–47]. ACSL4 catalyzes the conversion of PUFA into PUFA-CoA, a critical activation step in unsaturated fatty acid biosynthesis. Without ACSL4-mediated activation, free PUFA cannot enter the phospholipid pool, reducing membrane lipid peroxidation substrates and thereby inducing ferroptosis tolerance [48]. Previous studies indicate that HIF-1α overexpression enhances tumor cell resistance to ferroptosis [26] but whether it directly regulates the expression of lipid metabolism-related genes ACSL4 or LPCAT3 remains unclear. This study, through ChIP-seq validation sites and dual-luciferase site-directed mutagenesis, demonstrates that HIF-1α binds to the promoter region of ACSL4 to repress ACSL4 transcription. Given that HIF-1α typically functions as a transcriptional activator, we investigated whether HDACs contribute to the inhibition of chromatin accessibility in the HIF-1α–ACSL4 pathway. The results showed that TSA, a broad-spectrum HDAC inhibitor, restores hypoxia-induced ACSL4 mRNA suppression in a dose-dependent manner, indicating that HDACs are involved in this process. Furthermore, we found that it was HDAC3, not HDAC1, involved in HIF-1α-mediated ACSL4 transcriptional suppression according to RT–PCR and ChIP analysis (Fig. 4). In addition, we also discovered that ACSL4 overexpression restores PUFA-PL levels under hypoxia, reverses mitochondrial cristae protective structures, and lowers the IC_50_ of IKE (Fig. 5). Conversely, ACSL4 silencing recapitulates the HIF-1α overexpression phenotype. Notably, ACSL4 exhibits substrate preference, favoring AA utilization, suggesting that exogenous AA supplementation could further enhance the sensitization effect of ACSL4 overexpression.

PD-1 antibody immunotherapy is a revolutionary cancer treatment based on the principle of “immune checkpoint blockade” [50–54]. In this study, we found that PD-1 antibody monotherapy did not show significant efficacy in the ATC model. We observed that PX478 or ACSL4-OE combined with PD-1 significantly increased CD4⁺ T- and CD8⁺ T-cell infiltration and corresponding cytokine secretion (Fig. 6), leading to delayed tumor growth. To investigate the underlying mechanism by which ACSL4 overexpression enhances the antitumor immune efficacy of PD-1 therapy, we initially assessed dendritic cell maturation and antigen presentation. The results showed that the supernatant from ACSL4-overexpressing tumor cells significantly induced upregulation of CD80 and CD86 on dendritic cells, as well as elevated MHC-II levels. These indicators preliminarily suggest dendritic cell activation and enhanced antigen presentation, thereby providing a favorable condition for subsequent T cell-mediated antitumor responses.

On the basis of our results from the mouse xenograft model and cellular studies, HIF-1α and ACSL4 may be potential biomarker candidates for clinical stratification and treatment in patients with ATC. PX478, a small molecule inhibitor targeting HIF-1α, had been in phase I clinical trials for advanced solid tumors and lymphoma, yet these trials have been discontinued. Currently, it is primarily used in basic and preclinical research. However, the development of ACSL4 agonists to induce ferroptosis in tumor cells would be a key focus of our future research.

This study has several limitations. For instance, it relies on cellular models, animal experiments, and analysis of the TCGA database to elucidate the role of the HIF-1α–ACSL4 pathway in ferroptosis resistance in ATC. Owing to limitations in obtaining clinical samples, the correlation between HIF-1α and ACSL4 expression and its prognostic significance has not yet been systematically validated in the tissues of patients with ATC. Consequently, the conclusions are primarily based on basic laboratory experiments and require further validation in larger clinical cohorts. Furthermore, the animal experimental models were limited to subcutaneous tumors in nude mice and did not utilize in situ models, resulting in insufficient simulation of the immune microenvironment and the abnormal hypoxic vascular system.

## Conclusions

This study systematically elucidates a molecular mechanism by which HIF-1α directly binds to the ACSL4 promoter and represses its transcription, thereby interrupting the PUFA-PL synthesis pathway and diminishing lipid peroxidation substrates to confer ferroptosis resistance on ATC cells under hypoxic stress. The finding, for the first time, extends the transcriptional regulatory network of HIF-1α into ferroptosis-associated lipid metabolism and provides both theoretical and experimental rationales for precision therapeutic strategies targeting the HIF-1α–ACSL4 axis to potentiate ferroptosis sensitivity.

## Supplementary Information

Additional file 1.

Additional file 2.

Additional file 3.

Additional file 4.

Additional file 5.

Additional file 6.

Additional file 7.

Additional file 8.

Additional file 9.

## Data Availability

The datasets used and/or analyzed during the current study are available from the corresponding author on reasonable request.

## Abbreviations

ATC: Anaplastic thyroid carcinoma
HIF-1α: Hypoxia-inducible factor 1α
PUFA-PL: Polyunsaturated fatty acid phospholipids
ACSL4: Acyl-CoA synthase 4
LPCAT3: Lysophosphatidylcholine acyltransferase 3
GPX4: Glutathione peroxidase 4
PUFAs: Polyunsaturated fatty acids
HIFs: Hypoxia-inducible factors
FBS: Fetal bovine serum
P/S: Penicillin–streptomycin
SPF: Specific pathogen-free
ChIP: Chromatin immunoprecipitation
WT: Wild type
HE: Hematoxylin and eosin
PCA: Principal component analysis
PLS-DA: Partial least squares discriminant analysis
ANOVA: Analysis of variance
PI: Propidium iodide
