# Supplementary material for: HIF-1α enhances ferroptosis resistance in anaplastic thyroid carcinoma by suppressing ACSL4-mediated lipid metabolic homeostasis
Source: Cell Mol Biol Lett. 2026 Jun 27;31:132. doi: 10.1186/s11658-026-00973-1 (PMC13459170; doi:10.1186/s11658-026-00973-1)
Supplement: Supplementary file 1 [file 11658_2026_973_MOESM2_ESM.pdf]

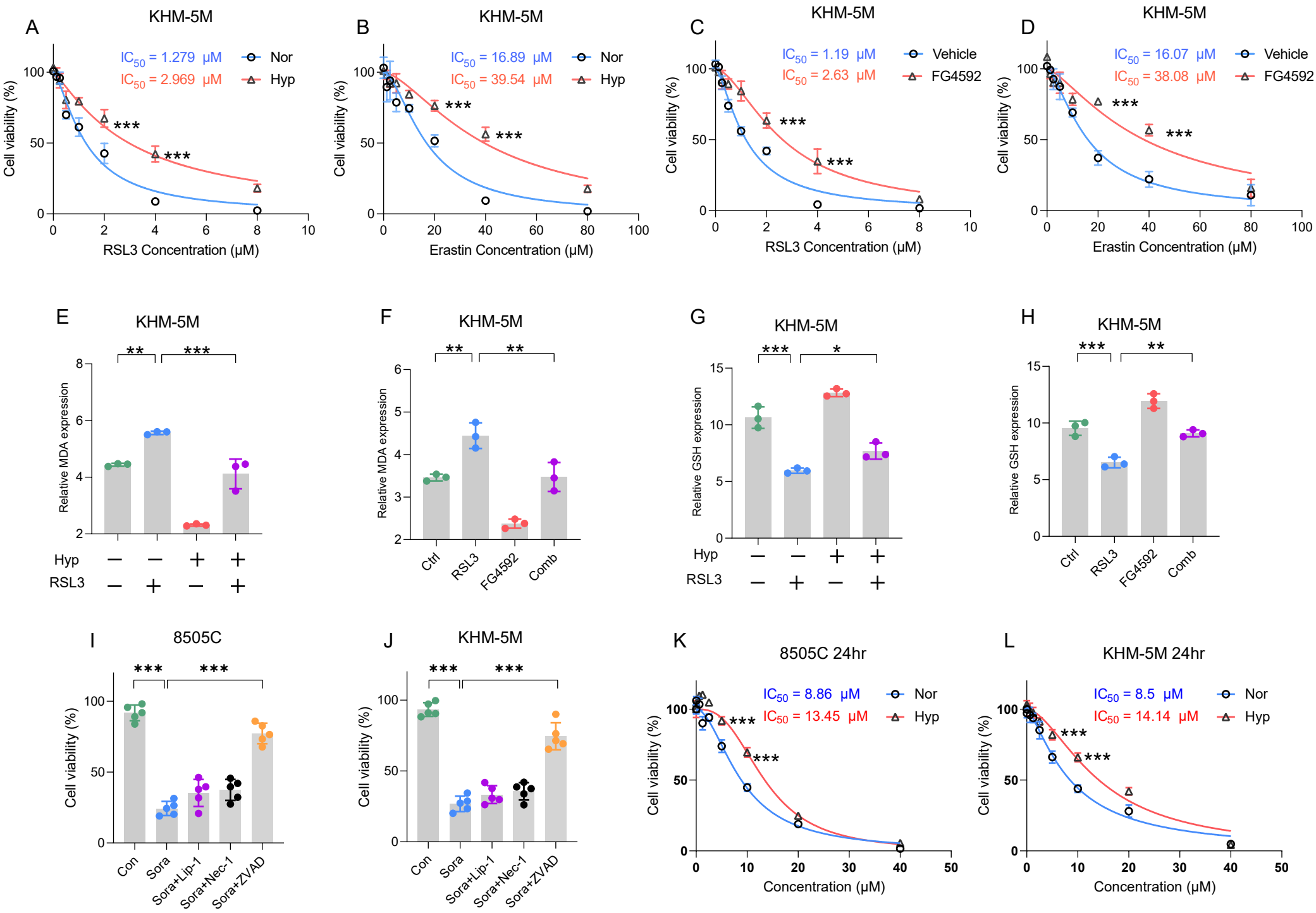
A
KHM-5M
IC_{50} = 1.279 μM
IC_{50} = 2.969 μM
Nor
Hyp
Cell viability (%)
RSL3 Concentration (μM)
B
KHM-5M
IC_{50} = 16.89 μM
IC_{50} = 39.54 μM
Nor
Hyp
Cell viability (%)
Erastin Concentration (μM)
C
KHM-5M
IC_{50} = 1.19 μM
IC_{50} = 2.63 μM
Vehicle
FG4592
Cell viability (%)
RSL3 Concentration (μM)
D
KHM-5M
IC_{50} = 16.07 μM
IC_{50} = 38.08 μM
Vehicle
FG4592
Cell viability (%)
Erastin Concentration (μM)
E
KHM-5M
Relative MDA expression
Hyp
RSL3
F
KHM-5M
Relative MDA expression
Ctrl
RSL3
FG4592
Comb
G
KHM-5M
Relative GSH expression
Hyp
RSL3
H
KHM-5M
Relative GSH expression
Ctrl
RSL3
FG4592
Comb
I
8505C
Cell viability (%)
Con
Sora
Sora+Lip-1
Sora+Nec-1
Sora+ZVAD
J
KHM-5M
Cell viability (%)
Con
Sora
Sora+Lip-1
Sora+Nec-1
Sora+ZVAD
K
8505C 24hr
IC_{50} = 8.86 μM
IC_{50} = 13.45 μM
Nor
Hyp
Cell viability (%)
Concentration (μM)
L
KHM-5M 24hr
IC_{50} = 8.5 μM
IC_{50} = 14.14 μM
Nor
Hyp
Cell viability (%)
Concentration (μM)
