# Supplementary material for: HIF-1α enhances ferroptosis resistance in anaplastic thyroid carcinoma by suppressing ACSL4-mediated lipid metabolic homeostasis
Source: Cell Mol Biol Lett. 2026 Jun 27;31:132. doi: 10.1186/s11658-026-00973-1 (PMC13459170; doi:10.1186/s11658-026-00973-1)
Supplement: Supplementary file 2 [file 11658_2026_973_MOESM3_ESM.pdf]

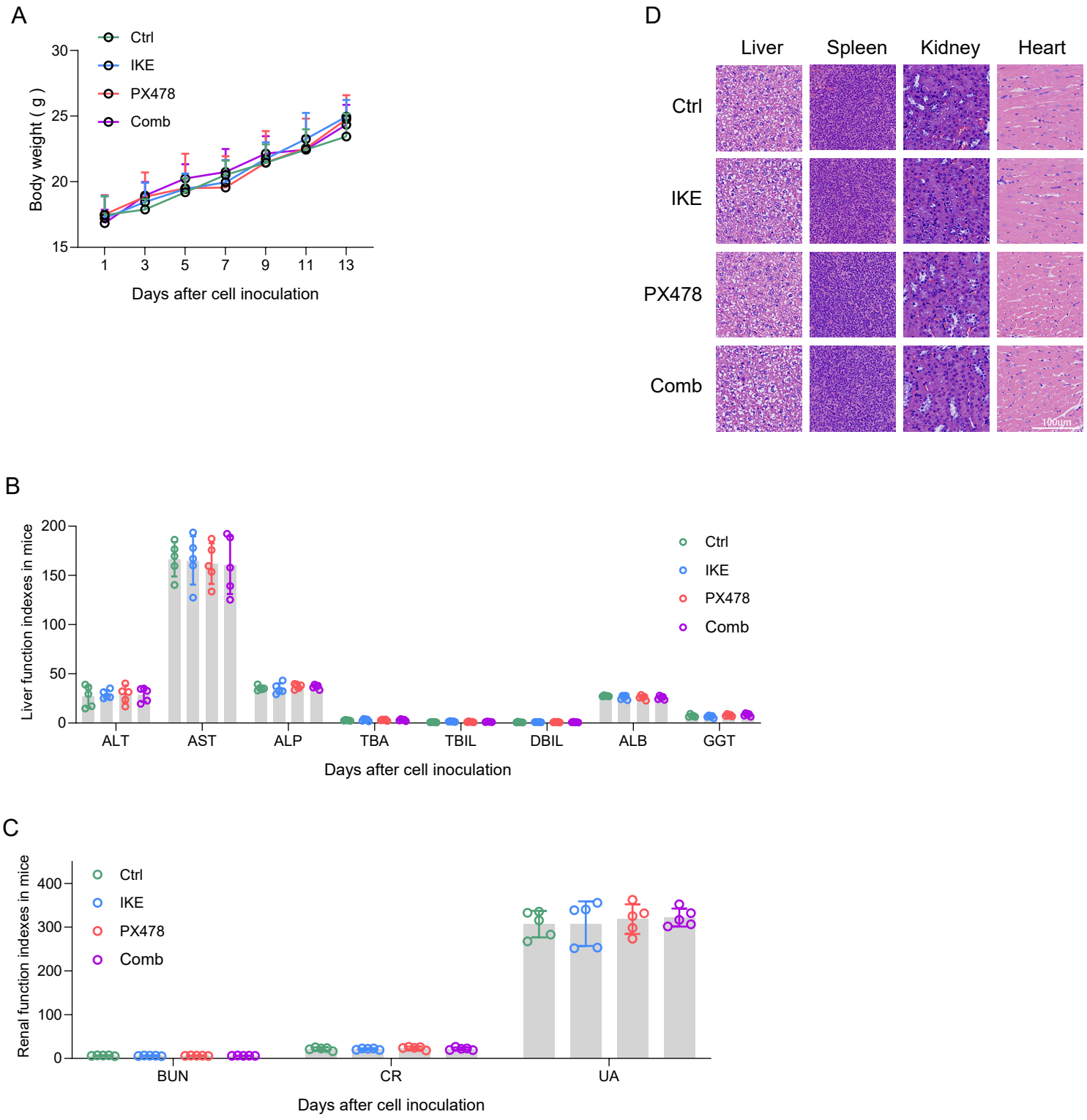
A
Ctrl
IKE
PX478
Comb
Body weight (g)
15
20
25
30
1
3
5
7
9
11
13
Days after cell inoculation
D
Liver
Spleen
Kidney
Heart
Ctrl
IKE
PX478
Comb
100μm
B
Liver function indexes in mice
0
50
100
150
200
ALT
AST
ALP
TBA
TBIL
DBIL
ALB
GGT
Days after cell inoculation
Ctrl
IKE
PX478
Comb
C
Renal function indexes in mice
0
100
200
300
400
Ctrl
IKE
PX478
Comb
BUN
CR
UA
Days after cell inoculation
