# Supplementary material for: HIF-1α enhances ferroptosis resistance in anaplastic thyroid carcinoma by suppressing ACSL4-mediated lipid metabolic homeostasis
Source: Cell Mol Biol Lett. 2026 Jun 27;31:132. doi: 10.1186/s11658-026-00973-1 (PMC13459170; doi:10.1186/s11658-026-00973-1)
Supplement: Supplementary file 3 [file 11658_2026_973_MOESM4_ESM.pdf]

A

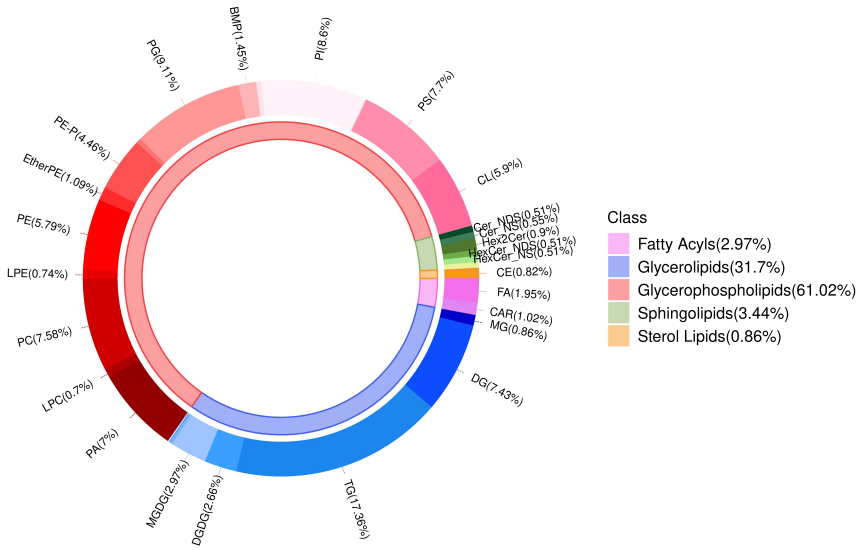
PI(8.6%)
PS(7.7%)
CL(5.9%)
Cer_NDS(0.51%)
Cer_NS(0.55%)
Hex2Cer(0.9%)
HexCer_NDS(0.51%)
HexCer_NS(0.51%)
CE(0.82%)
FA(1.95%)
CAR(1.02%)
MG(0.86%)
DG(7.43%)
TG(17.36%)
DGDG(2.66%)
MGDG(2.97%)
PA(7%)
LPC(0.7%)
PC(7.58%)
LPE(0.74%)
PE(5.79%)
EtherPE(1.09%)
PE-P(4.46%)
PG(9.11%)
BMP(1.45%)
Class
Fatty Acyls(2.97%)
Glycerolipids(31.7%)
Glycerophospholipids(61.02%)
Sphingolipids(3.44%)
Sterol Lipids(0.86%)

B

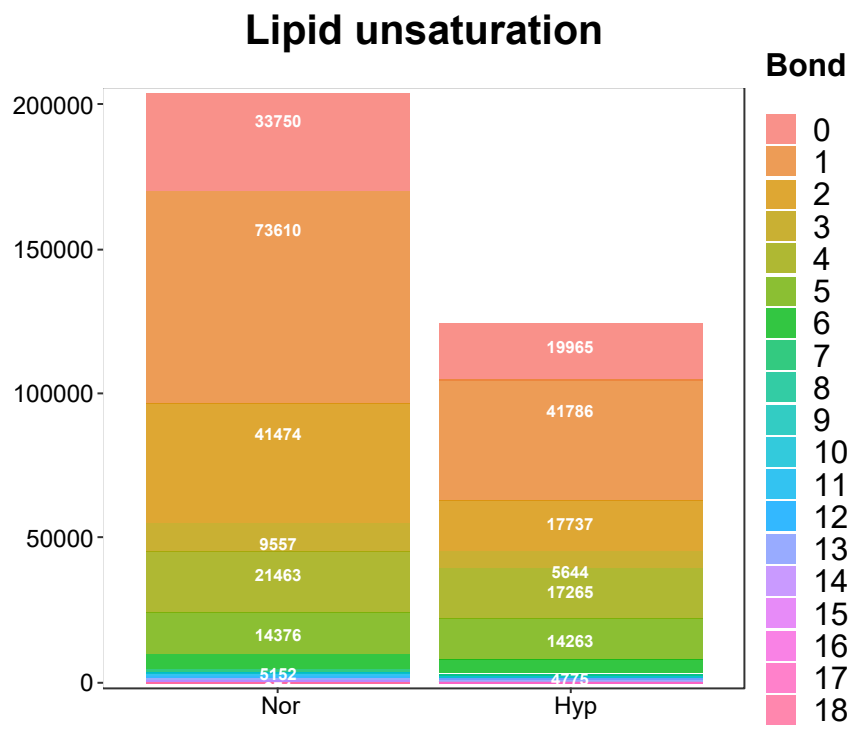
Lipid unsaturation
Bond
0
1
2
3
4
5
6
7
8
9
10
11
12
13
14
15
16
17
18
200000
150000
100000
50000
0
33750
73610
41474
9557
21463
14376
5152
19965
41786
17737
5644
17265
14263
4775
Nor
Hyp

C

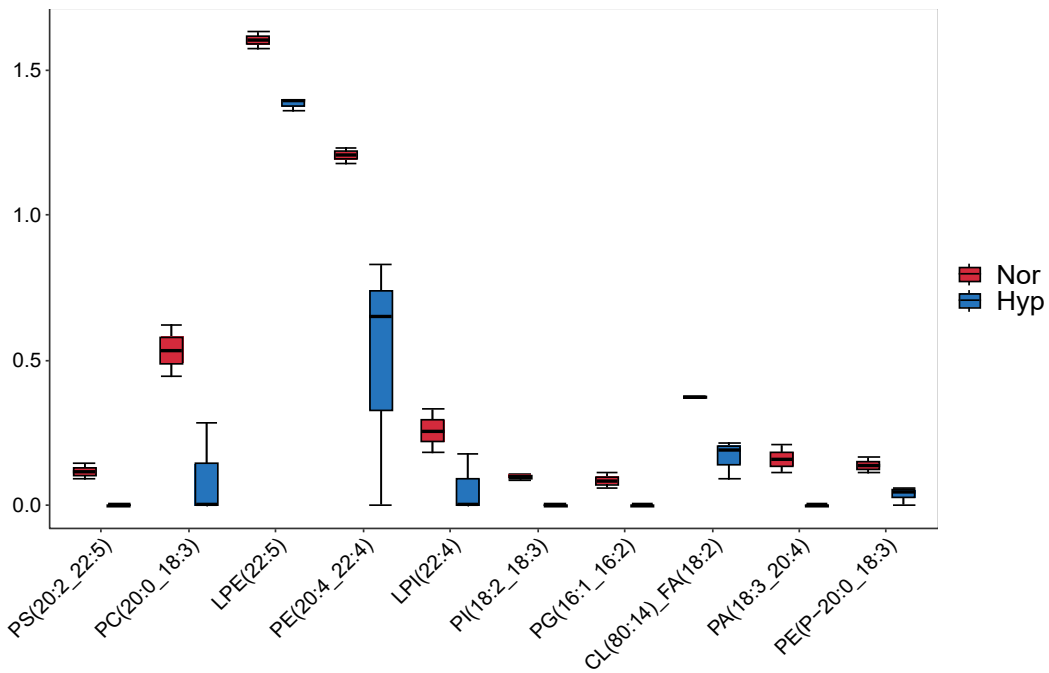
1.5
1.0
0.5
0.0
Nor
Hyp
PS(20:2_22:5)
PC(20:0_18:3)
LPE(22:5)
PE(20:4_22:4)
LPI(22:4)
PI(18:2_18:3)
PG(16:1_16:2)
CL(80:14)_FA(18:2)
PA(18:3_20:4)
PE(P-20:0_18:3)
