# Supplementary material for: HIF-1α enhances ferroptosis resistance in anaplastic thyroid carcinoma by suppressing ACSL4-mediated lipid metabolic homeostasis
Source: Cell Mol Biol Lett. 2026 Jun 27;31:132. doi: 10.1186/s11658-026-00973-1 (PMC13459170; doi:10.1186/s11658-026-00973-1)
Supplement: Supplementary file 4 [file 11658_2026_973_MOESM5_ESM.pdf]

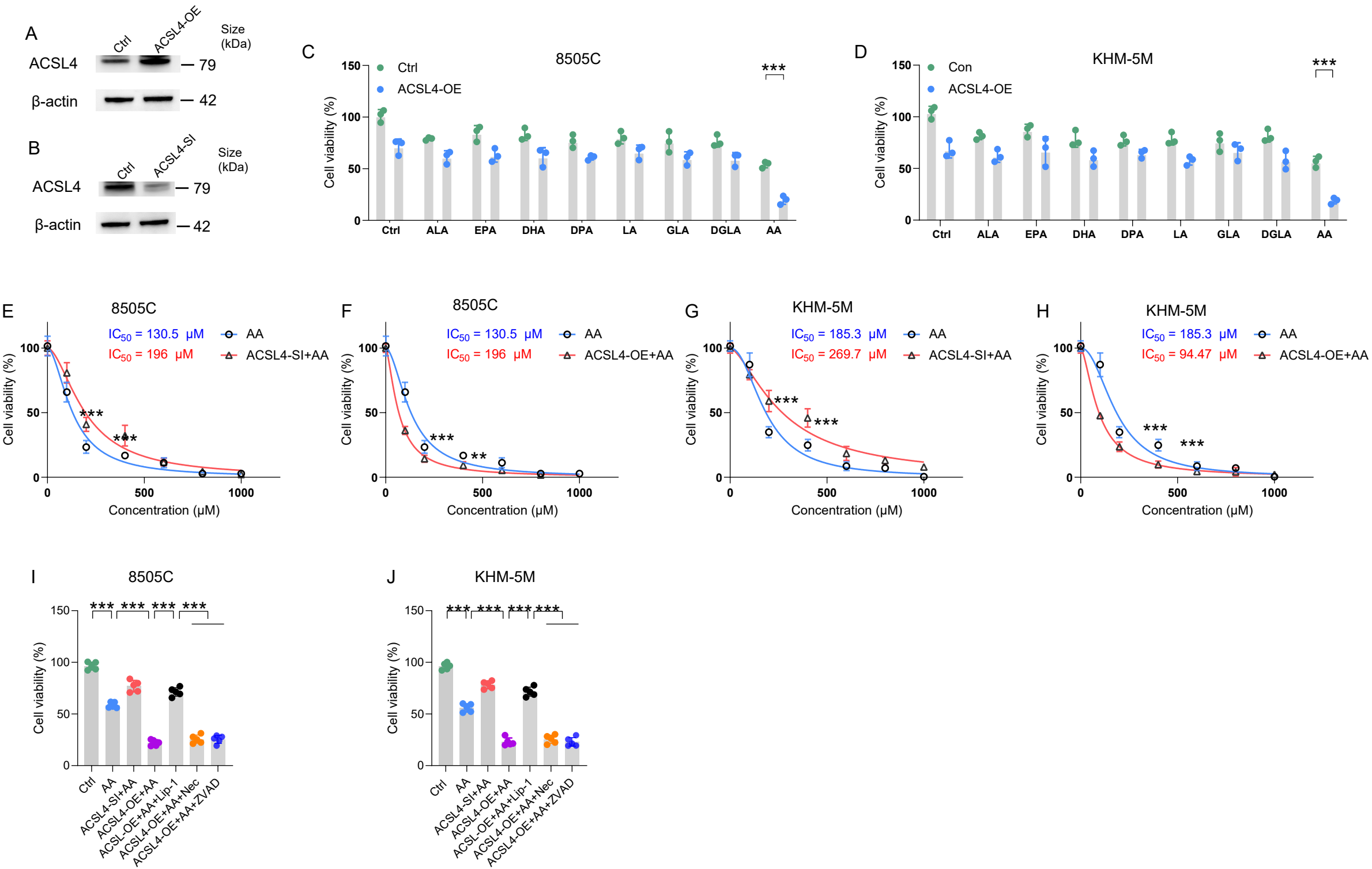
A
Ctrl
ACSL4-OE
Size (kDa)
ACSL4
β-actin
79
42
B
Ctrl
ACSL4-SI
Size (kDa)
ACSL4
β-actin
79
42
C
8505C
Ctrl
ACSL4-OE
Cell viability (%)
Ctrl ALA EPA DHA DPA LA GLA DGLA AA
***
D
KHM-5M
Con
ACSL4-OE
Cell viability (%)
Ctrl ALA EPA DHA DPA LA GLA DGLA AA
***
E
8505C
IC50 = 130.5 μM
IC50 = 196 μM
AA
ACSL4-SI+AA
Cell viability (%)
Concentration (μM)
F
8505C
IC50 = 130.5 μM
IC50 = 196 μM
AA
ACSL4-OE+AA
Cell viability (%)
Concentration (μM)
G
KHM-5M
IC50 = 185.3 μM
IC50 = 269.7 μM
AA
ACSL4-SI+AA
Cell viability (%)
Concentration (μM)
H
KHM-5M
IC50 = 185.3 μM
IC50 = 94.47 μM
AA
ACSL4-OE+AA
Cell viability (%)
Concentration (μM)
I
8505C
Cell viability (%)
Ctrl
AA
ACSL4-SI+AA
ACSL4-OE+AA
ACSL-OE+AA+Lip-1
ACSL4-OE+AA+Nec
ACSL4-OE+AA+ZVAD
J
KHM-5M
Cell viability (%)
Ctrl
AA
ACSL4-SI+AA
ACSL4-OE+AA
ACSL-OE+AA+Lip-1
ACSL4-OE+AA+Nec
ACSL4-OE+AA+ZVAD
