# Supplementary material for: HIF-1α enhances ferroptosis resistance in anaplastic thyroid carcinoma by suppressing ACSL4-mediated lipid metabolic homeostasis
Source: Cell Mol Biol Lett. 2026 Jun 27;31:132. doi: 10.1186/s11658-026-00973-1 (PMC13459170; doi:10.1186/s11658-026-00973-1)
Supplement: Supplementary file 5 [file 11658_2026_973_MOESM6_ESM.pdf]

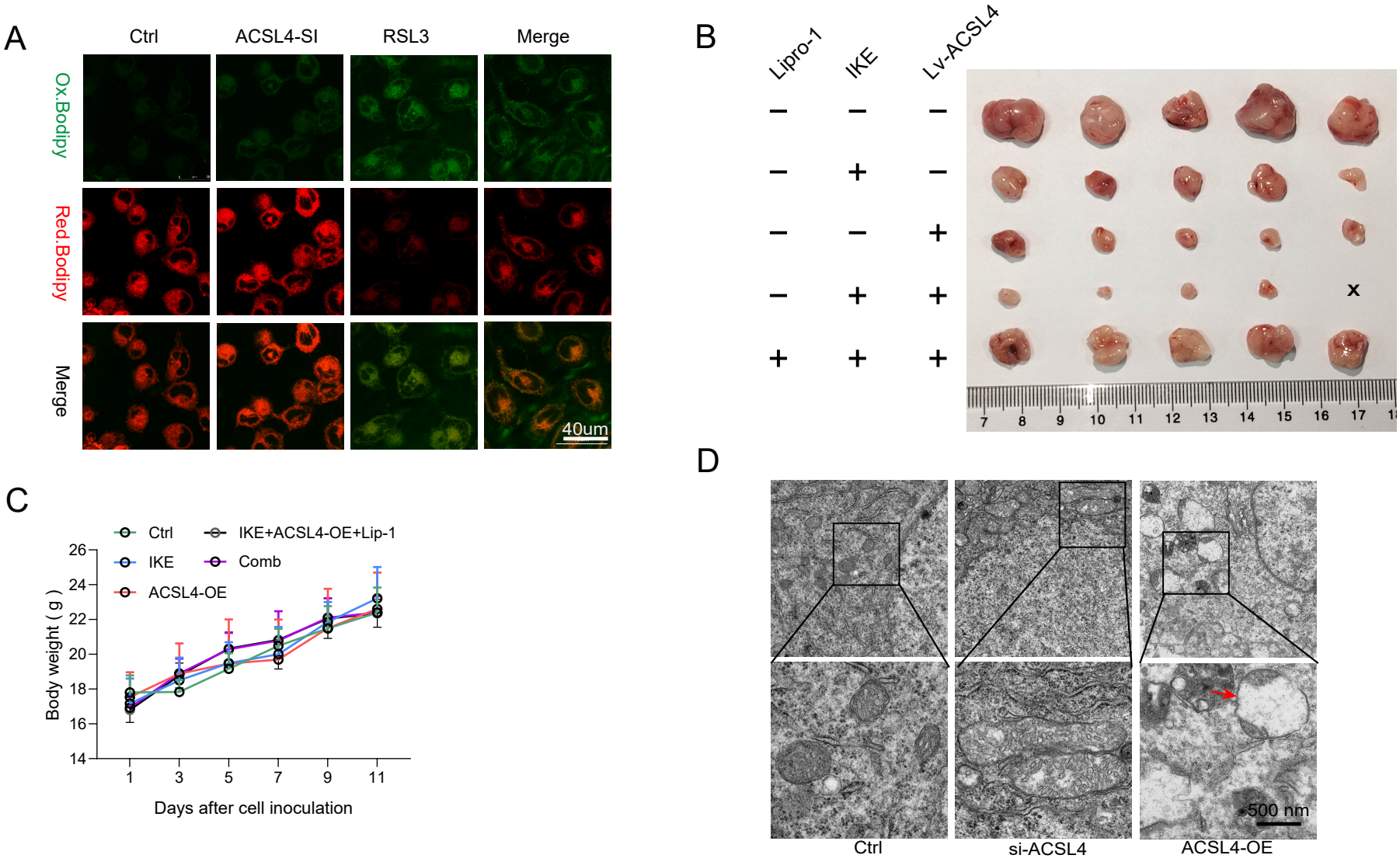
A
Ctrl
ACSL4-SI
RSL3
Merge
Ox.Bodipy
Red.Bodipy
Merge
40um
B
Lipro-1
IKE
Lv-ACSL4
x
C
Ctrl
IKE
ACSL4-OE
IKE+ACSL4-OE+Lip-1
Comb
Body weight (g)
Days after cell inoculation
D
Ctrl
si-ACSL4
ACSL4-OE
500 nm
