# Supplementary material for: HIF-1α enhances ferroptosis resistance in anaplastic thyroid carcinoma by suppressing ACSL4-mediated lipid metabolic homeostasis
Source: Cell Mol Biol Lett. 2026 Jun 27;31:132. doi: 10.1186/s11658-026-00973-1 (PMC13459170; doi:10.1186/s11658-026-00973-1)
Supplement: Supplementary file 6 [file 11658_2026_973_MOESM7_ESM.pdf]

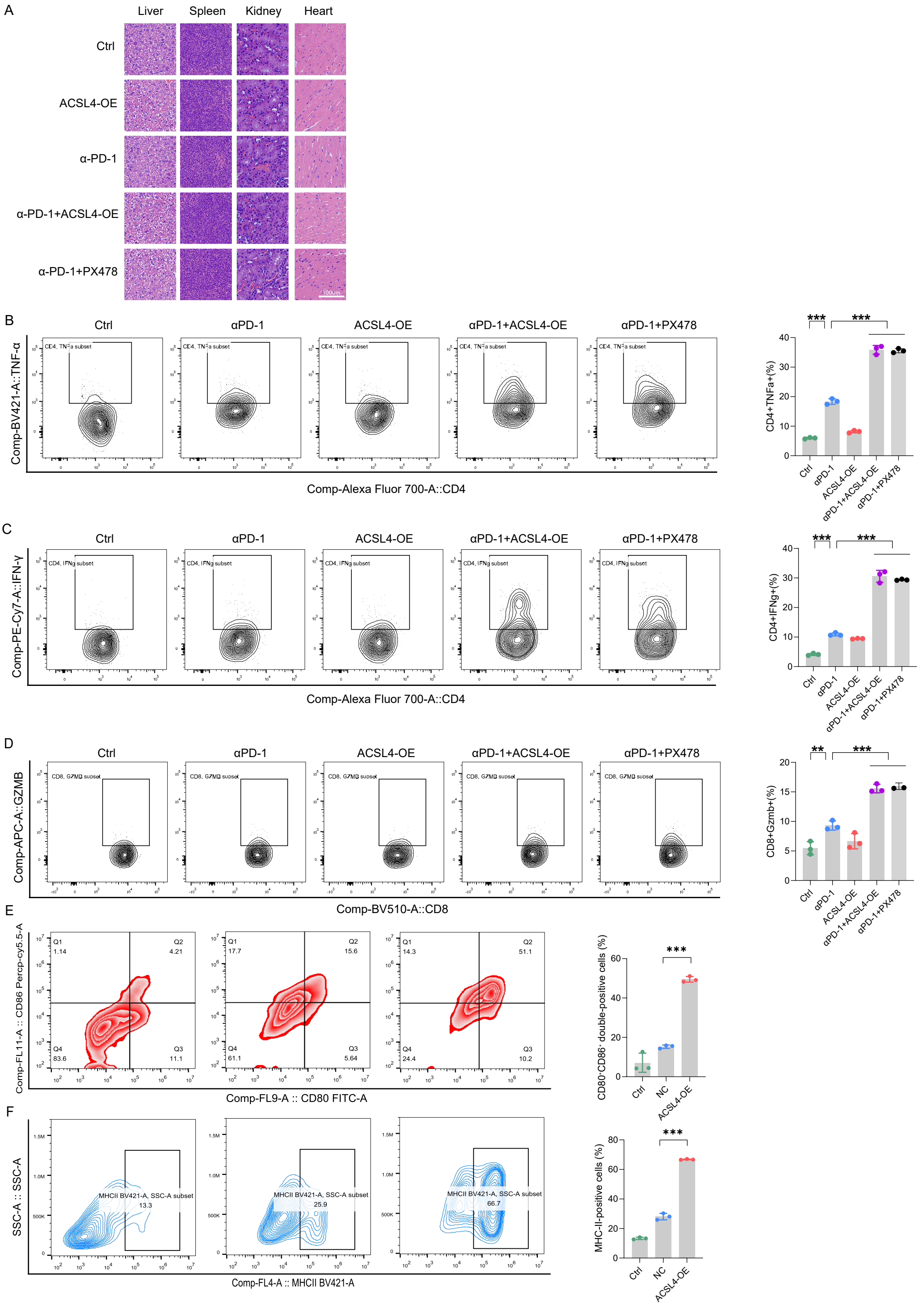
A
Liver
Spleen
Kidney
Heart
Ctrl
ACSL4-OE
α-PD-1
α-PD-1+ACSL4-OE
α-PD-1+PX478
100um
B
Ctrl
αPD-1
ACSL4-OE
αPD-1+ACSL4-OE
αPD-1+PX478
Comp-BV421-A::TNF-α
Comp-Alexa Fluor 700-A::CD4
CD4+TNFa+(%)
C
Comp-PE-Cy7-A::IFN-γ
CD4+IFNg+(%)
D
Comp-APC-A::GZMB
Comp-BV510-A::CD8
CD8+Gzmb+(%)
E
Comp-FL11-A :: CD86 Percp-cy5.5-A
Comp-FL9-A :: CD80 FITC-A
Q1 1.14
Q2 4.21
Q4 83.6
Q3 11.1
Q1 17.7
Q2 15.6
Q4 61.1
Q3 5.64
Q1 14.3
Q2 51.1
Q4 24.4
Q3 10.2
CD80+CD86+ double-positive cells (%)
Ctrl
NC
ACSL4-OE
F
SSC-A :: SSC-A
Comp-FL4-A :: MHCII BV421-A
MHCII BV421-A, SSC-A subset
13.3
25.9
66.7
MHC-II-positive cells (%)
