# Supplementary material for: HIF-1α enhances ferroptosis resistance in anaplastic thyroid carcinoma by suppressing ACSL4-mediated lipid metabolic homeostasis
Source: Cell Mol Biol Lett. 2026 Jun 27;31:132. doi: 10.1186/s11658-026-00973-1 (PMC13459170; doi:10.1186/s11658-026-00973-1)

Fig1E

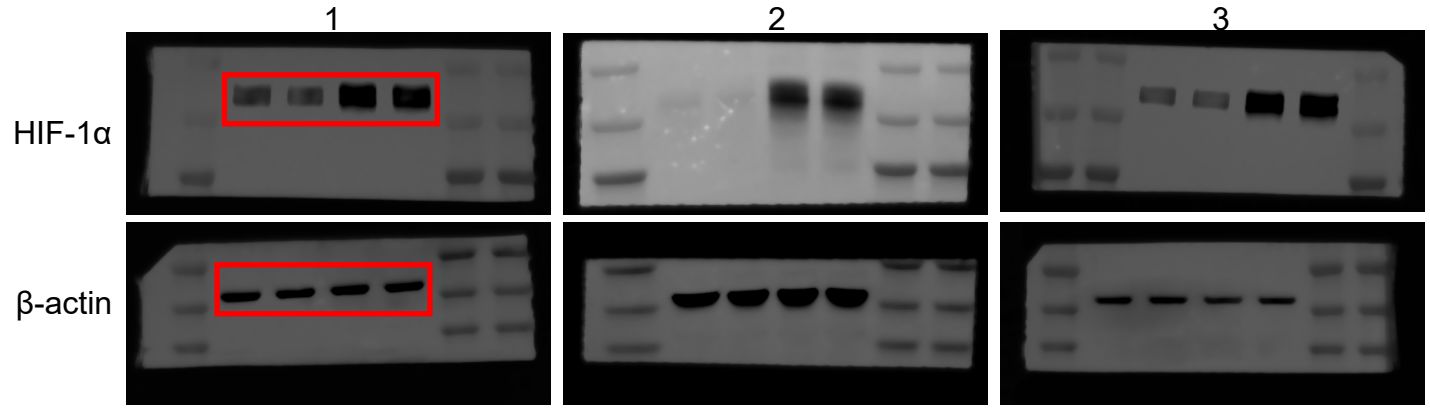

SFig4A

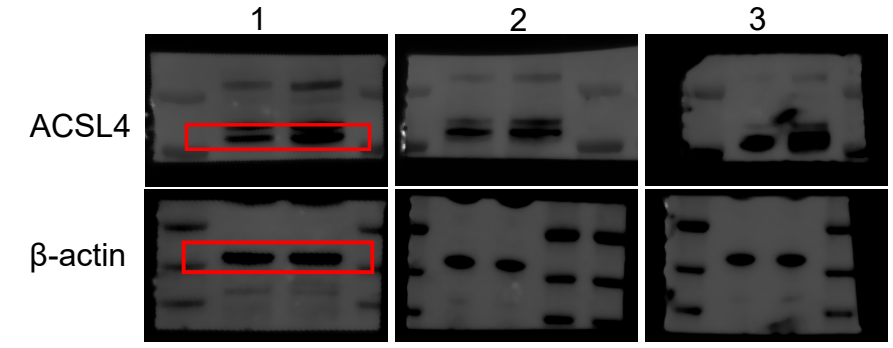

Fig2F

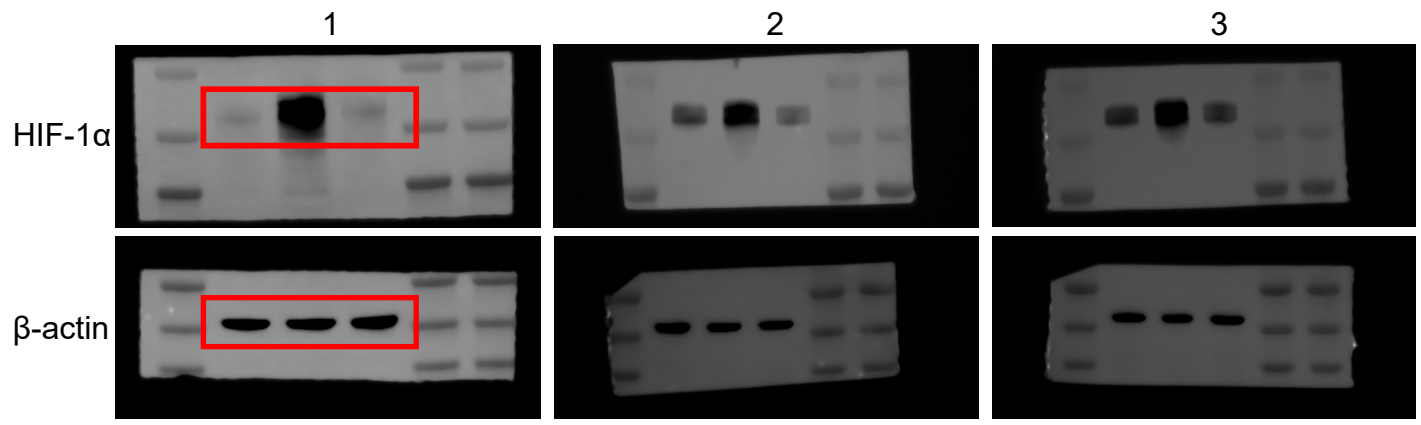

SFig4B

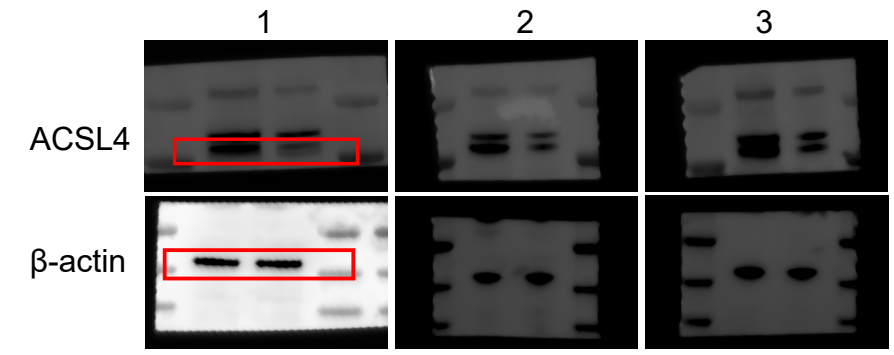

Fig2G

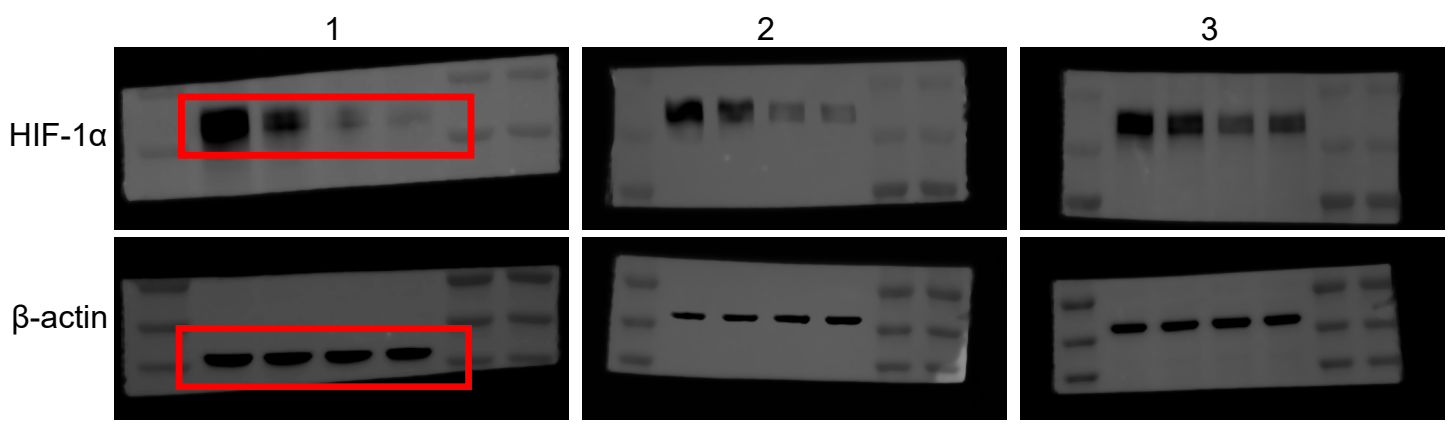

Fig4B

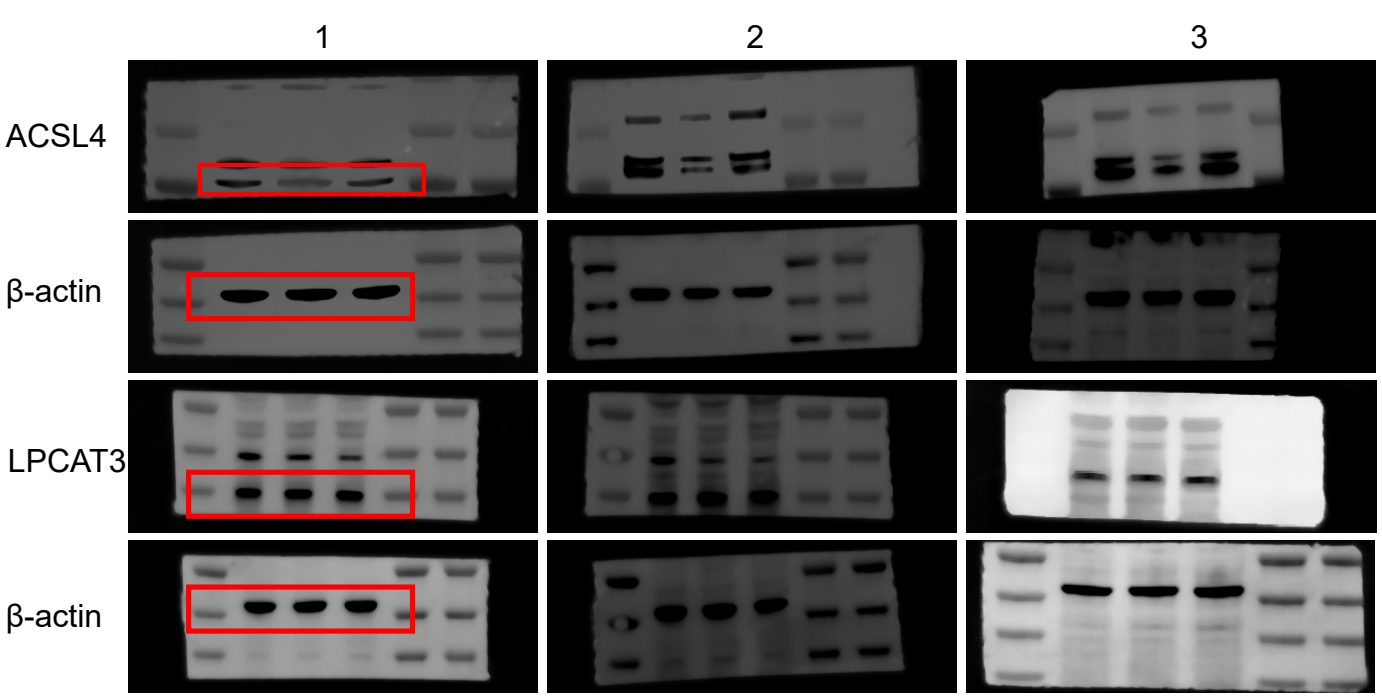

Supplement: Supplementary file 7 [file 11658_2026_973_MOESM9_ESM.pdf]
