# Supplementary material for: HIF-1α enhances ferroptosis resistance in anaplastic thyroid carcinoma by suppressing ACSL4-mediated lipid metabolic homeostasis
Source: Cell Mol Biol Lett. 2026 Jun 27;31:132. doi: 10.1186/s11658-026-00973-1 (PMC13459170; doi:10.1186/s11658-026-00973-1)
Supplement: Supplementary file 8 [file 11658_2026_973_MOESM8_ESM.docx]

**Supplementary Figure Legends**

**SFig1 Hypoxia drives ATC cells' resistance to ferroptosis. A-D**: Exposure to 1% oxygen (24 h) or the chemical inducer FG-4592 (50 mM, 24 h) reduced the drug sensitivity of ATC cells to erastin (10 μM, 24 h)/RSL3 (1 μM, 24 h). **E, F**: Exposure to 1% oxygen (H) or chemically induced hypoxia (I) attenuates the increase in intracellular malondialdehyde (MDA) levels in ATC cells induced by the ferroptosis inducer RSL3 (1 μM, 24 h)(KHM-5M).**G, H**: 1% oxygen environment (J) or chemically induced hypoxia (K) reversed the downregulation of intracellular GSH levels in ATC cells induced by the ferroptosis inducer RSL3 (1 μM, 24 h)(KHM-5M).**I, J**: Cell viability of 8505C and KHM-5M cells treated with Sora(10 μM,24 h), Sora+ Lip-1(0.1 μM), Sora + Nec-1(1 μM), or Sora + ZVAD(10 μM).**K, L**: Dose-response curves and IC₅₀ values for sorafenib in 8505C and KHM-5M cells under normoxic (Nor) and hypoxic (Hyp) conditions.

**SFig2 HIF-1α facilitates the development of ATC tumors. A-C**: No significant differences in body weight were observed among groups throughout the experiment. Multi-marker serum assays revealed no differences between experimental and blank control groups.**D**: HE staining of major organs in nude mice showed no evident pathological damage in sectioned specimens across all groups.

**SFig3 Hypoxia inhibits tumor cell ferroptosis by suppressing lipid peroxidation accumulation. A**:Component analysis of the lipidome in ATC cells. **B**: Analysis of glycerophospholipid unsaturation levels at different oxygen concentrations. **C**: Box plot illustrating the downregulated PUFA-PL.

**SFig4** **HIF-1α suppresses ferroptosis by regulating ACSL4. A, B**: Protein validation of siACSL4 and ACSL4-OE cells in ATC. **C, D**: Bar chart of cell viability in ACSL4-OE versus Con ATC cells treated with various polyunsaturated fatty acids. **E-H**: Sensitivity of ATC cells to arachidonic acid under different ACSL4 expression conditions. **I, J**: Survival rates of 8505C and KHM-5M cells treated with AA and various inhibitors under conditions of ACSL4 knockdown (ACSL4-SI) or overexpression (ACSL4-OE).

**SFig5** **Overexpression of ACSL4 abrogates the ferroptosis resistance conferred by HIF-1α. A**: Confocal imaging revealed green fluorescence representing lipid peroxidation-positive signals. Hypoxia and siACSL4 attenuated green fluorescence, whereas ACSL4-OE restored fluorescence intensity by counteracting hypoxia effects. **B**: Post-experiment tumor removal and photography visually demonstrate therapeutic outcomes.**C**: Mouse body weight monitoring during the experiment showed no significant differences between groups.**D**: Electron micrographs of mitochondria in ATC cells treated with Ctrl, si-ACSL4, and ACSL4-OE.

**SFig6** **PX478 enhances PD-1 blockade efficacy by promoting tumor-infiltrating T cell immune responses. A**: Hematoxylin and eosin (HE) stained sections of major organs from C57 mice.**B**: Flow cytometry quantitative bar chart of TNF-α^+^CD4^+^ T cell proportion within C57 mice ATC-transplanted tumors.**C**: Flow cytometry quantitative bar chart showing the proportion of IFN-γ^+^CD4^+^ T cells within C57 mice's ATC-transplanted tumors.**D**: Flow cytometry quantitative bar chart showing the proportion of Gzmb^+^CD8^+^ T cells within C57 mice's ATC-transplanted tumors.**E, F**: Flow cytometric analysis of CD80⁺CD86⁺ double-positive and MHC-II-positive antigen-presenting cells in the blank control group, NC group, and ACSL4-OE group.
